# Supplementary material for: Dynamics of diversified A-to-I editing in Streptococcus pyogenes is governed by changes in mRNA stability
Source: Nucleic Acids Res. 2024 Aug 1;52(18):11234–53. doi: 10.1093/nar/gkae629 (PMC11472039; doi:10.1093/nar/gkae629)
Supplement: gkae629_Supplemental_Files [file gkae629_supplemental_files.zip › 240629_Manuscript_Supplement.pdf]

Supplementary Material

Dynamics of diversified A-to-I editing in *Streptococcus pyogenes* is governed by changes in mRNA stability

Thomas F. Wulff[1], Karin Hahnke[1], Anne-Laure Lécrivain[1], Katja Schmidt[1], Rina Ahmed-Begrich[1], Knut Finstermeier[1] and Emmanuelle Charpentier[1,2,*]

[1] Max Planck Unit for the Science of Pathogens, 10117 Berlin, Germany
[2] Institute for Biology, Humboldt University Berlin, 10115 Berlin, Germany

* To whom correspondence should be addressed. Email: research@emmanuelle-charpentier.org.

SUPPLEMENTARY METHODS 2
SUPPLEMENTARY FIGURES 9
SUPPLEMENTARY TABLES 24
SUPPLEMENTARY TABLE LEGENDS 39
SUPPLEMENTARY REFERENCES 41

SUPPLEMENTARY METHODS

Construction of genomic mutants

General approach. For anhydrotetracycline (AHT)-inducible gene expression, we used the previously described Tn*10*-derived P_{tet} cassette, harbouring the transcriptional repressor *tetR* with two divergent promoters and three operator sites (hereafter referred to simply as P_{tet}), together with *cat86* as antibiotic marker (1, 2), and integrated the cassette into the *S. pyogenes* genome using suicide vectors. The *cat86*-P_{tet} cassette was obtained from pEC536 (2) by restriction digestion with PstI/SmaI and ligated into PstI/SmaI-digested pEC801 to obtain pEC808. An internal NdeI restriction site in *tetR* was synonymously mutated by site-directed mutagenesis of pEC808 using OLEC3322/3323 to obtain pEC812 with *tetR*(A582C).

Deletion of tadA. The up- and downstream flanking regions of *tadA* were amplified from SF370 genomic DNA using the primers listed in Table S2. The flanking regions were then PCR-ligated to the lox71-$P_{ermAM/B}$-*ermAM/B*-lox66 cassette and integrated into pEC801 using Gibson Assembly® Master Mix generating pEC2899.

Inducible expression of RNase J1. The up- and downstream flanking regions of *rnjA* were amplified from SF370 genomic DNA using the primers listed in Table S2. The flanking regions were then sequentially integrated into pEC812 by restriction-ligation using PstI (upstream) and NdeI (downstream) generating pEC852.

Inducible expression of editing target gene operons. The *cat86*-P_{tet} cassette was PCR-amplified from pEC808 and assembled into pEC801 with a terminator upstream of *cat86* and flanked by two Bsp1407I restriction sites using Gibson Assembly® Master Mix resulting in pEC2901. Regions upstream and downstream of the operon transcriptional start site (TSS) were then amplified by Phusion PCR from SF370 wildtype genomic DNA using the primers listed in Table S2 and introduced into Bsp1407I-digested pEC2901 using Gibson Assembly® Master Mix generating pEC2964, pEC2965 and pEC2966.

Inducible expression of TadA. The TT_{tadA}-*cat86*-P_{tet} cassette was integrated between *SPy_0208* and *tadA*, which together constitute a bicistronic operon. As a control, the TT_{tadA}-*cat86*-P_{tet} cassette was integrated directly downstream of the *tadA* coding sequence. The terminator region and the up- and downstream flanking regions were PCR-ligated to the *cat86*-P_{tet}

cassette using the primers listed in Table S2. The purified amplicon was introduced in the PCR-linearised suicide vector pEC801 using Gibson Assembly® Master Mix, resulting in pEC2913 and pEC2914. Due to promoter leakage, the ribosome binding site was adapted through site-directed mutagenesis using OLEC13498/13499 or OLEC13498/13500, generating pEC3000 from pEC2913 and pEC3001 from pEC2914, respectively. *tadA* expression was further optimised by site-directed mutagenesis of pEC3001 using OLEC13766/13767, generating pEC3021.

Electroporation, selection and validation of strains. Plasmids were linearised by digestion with Cfr42I and introduced into electro-competent *S. pyogenes* SF370 by electroporation (3). Strains were selected for two passages on TSA plates with 3% blood and 6 µg/mL chloramphenicol, cultured in THY without antibiotics, and flash-frozen in liquid nitrogen in THY with 20% glycerol. All strains were validated by Sanger sequencing using gene-specific and *cat86*-P_{tet} cassette-targeting primers as well as primers targeting the virulence regulators *mga*, *covRS* and *ropB* (see Table S2), which are common mutational hotspots affecting *S. pyogenes* virulence (4). A schematic overview of the modified loci is shown in Figure S1.

Construction of plasmids

Templates for tRNA in vitro *transcription.* tRNA template and T7 promoter sequences were assembled from six oligonucleotides in a modular approach using the HindIII and BamHI restriction sites in pUC19 as previously described (5). For *S. pyogenes* tRNAs, the CCA terminus with an overlapping MvaI restriction site was artificially added to the 3′ end of the tRNA gene. In brief, oligonucleotides were 5′ phosphorylated, annealed and ligated into BamHI-/HindIII-digested pUC19. Mutations in the T7 promoter of pEC2405 and pEC2406 were repaired by site-directed mutagenesis using primers OLEC9115/9117 for pEC2405 and OLEC9115/9116 for pEC2406, respectively.

TadA protein purification. The *S. pyogenes tadA* gene (NC_002737.2:187,764..188,279) was PCR-amplified from *S. pyogenes* SF370 genomic DNA using primers OLEC8780/8782 and the *E. coli tadA* gene was amplified from *E. coli* NEB® 5-alpha genomic DNA using OLEC8956/8958. pET-21a(+) and PCR amplicons were digested with FastDigest NdeI and FastDigest HindIII,

ligated and introduced by transformation into *E. coli*, generating plasmids pEC2360 (*S. pyogenes tadA*) and pEC2389 (*E. coli tadA*).

Ectopic expression of tadA *in S. pyogenes.* The constitutive promoter P_{gyrA} from *S. agalactiae* was amplified from pEC455 using OLEC11695/11697. *tadA* was amplified from genomic DNA using OLEC11698/11541, and a C-terminal His_6 tag was added by PCR-mediated ligation to the assembled oligos OLEC11059/11060 using the primers OLEC11692/11698. Amplicons were assembled in OLEC11689/11694-amplified pEC2173 by Gibson assembly, generating pEC2813. The empty vector control was built by Gibson assembly of OLEC11695/11696-amplified P_{gyrA} from pEC455 into PCR-amplified pEC2173 generating pEC2812.

ermBL-based reporter assay. A schematic overview of the reporter construct is shown in Figure S11. The *ermB* leader peptide (*ermBL*)-containing 5′ UTR of Tn*917 ermB*, 30 nt of the *ermB* CDS, the *ftsK*-derived A-to-I editing site and a Gly_3Ser linker were amplified using OLEC14017/14018 from the assembled oligos OLEC14013 to OLEC14016. The firefly luciferase *ffluc* was amplified from pEC2173 using OLEC14009/14010, and the terminator TT3 was assembled using OLEC14011/14012. All amplicons were ligated by PCR using OLEC13810/14017 and integrated into PCR-amplified pEC2812 using OLEC10676/14008 using Gibson Assembly® Master Mix generating pEC3045. The *ermBL*(M1*) mutant reporter pEC3046 was generated by site-directed mutagenesis using OLEC14019/14020. The mutant reporter pEC3047 with a deletion of *ermBL* and the intergenic region between *ermBL* and *ermB* was constructed with OLEC14135/14136-amplified pEC3045 and assembled oligos OLEC14163/14164 using Gibson Assembly® Master Mix. The start codon mutant of pEC3047 (*i.e.*, pEC3069) was generated by site-directed mutagenesis using OLEC14216/14217.

Quantitative RT-PCR

Samples from bacterial cultures were collected by addition of an equal volume of ice-cold acetone/ethanol (1:1, v/v), washed using TE-sucrose (50 mM Tris-HCl, 10 mM EDTA, 50 mM NaCl, 25% sucrose, pH 8.0), and stored at -80°C, if necessary. Cells were lysed enzymatically, and RNA was extracted using TRIzol / chloroform as described in Material and Methods. 10 µg of total RNA were treated with 1 µL of TURBO DNase (Invitrogen) for 30 min at 37°C in 50 µL

of 1x TURBO DNase buffer, before adding another 1 µL of TURBO DNase and incubating reactions for further 30 min. Reactions were stopped using Inactivation Reagent as described by the manufacturer, and DNase-treated RNA was purified using RNA Clean & Concentrator-5 (Zymo Research). RNA quantity and quality were assessed using a NanoDrop One UV-Vis Spectrophotometer, and RNA integrity was verified by agarose gel electrophoresis. The absence of DNA was verified by performing RT-PCR without addition of reverse transcriptase as described in Material and Methods for the detection of editing levels by Sanger sequencing. In brief, RNA was reverse transcribed in a 20 µL reaction using random hexamer primers (Thermo Scientific) with and without SuperScript III Reverse Transcriptase (Invitrogen), and 1 µL of the reaction was used as input in a 50 µL PCR reaction using recombinant *Taq* DNA Polymerase (Invitrogen) and editing target gene-specific primers.

10 ng of DNase-treated total RNA were consequently used as qRT-PCR template in a 20 µL reaction using the Power SYBR Green RNA-to-CT 1-Step Kit (Applied Biosystems) with primers at 100 nM as described by the manufacturer. Reactions were run in a MicroAmp™ Fast Optical 96-Well Reaction Plate (Applied Biosystems) in a QuantStudio 5 Real-Time PCR System (Applied Biosystems) using standard cycling mode with the following cycling conditions: reverse transcription for 30 min at 48°C, enzyme inactivation for 10 min at 95°C, and 40 cycles consisting of 15 s at 95°C and 1 min at 60°C each. All samples were analysed with at least two technical replicates and three biological replicates. C_q values were determined using the QuantStudio Design & Analysis Software, and target gene expression was calculated as previously described (6, 7):

$$relative\ target\ gene\ expression = \frac{{E_{target}}^{\Delta Cq_{target}}}{\sqrt[n]{\prod_{i=1}^{n} {E_{reference,i}}^{\Delta Cq_{reference,i}}}}$$

Primers for qRT-PCR were designed using Primer-BLAST (8) according to the recommendations of the Power SYBR Green RNA-to-CT 1-Step Kit (Applied Biosystems) generating amplicons of 80 to 125 bp length (compare Table S4). Primers were required to have a melting temperature (T_m) between 58°C and 62°C, a maximum T_m difference of 2°C, a length of 18 to 22 nt, a GC content between 40% and 60%, and a maximum number of 2 G or C bases in the last 5 bases at the primer’s 3′ end. Primers were obtained from Sigma-Aldrich (standard desalt purification), and sequences are listed in Table S2. *gyrA*, *rpoB*, *era*, and *secA*

were selected as reference genes based on a recent publication (9), and 16S rRNA was used as reference gene for RNA stability measurements. Primer efficiencies were obtained from $\log_{10}$ dilution series (10 ng to 0.01 ng RNA) from three biological replicates with technical duplicates each. RNA from EC2224 transformed with pEC3045 and grown to mid-logarithmic growth phase in the presence of 0 ng/mL AHT was used for all genes but *slo*, for which RNA from EC3453 grown to mid-logarithmic growth phase in the presence of 100 ng/mL AHT was used. C_q values were plotted against the $\log_{10}$ of the input RNA amount (Figure S2), and linear regression was performed ($R^2 > 0.98$). Efficiencies were then calculated from the average slope of the three biological replicates with PCR efficiency $E = 10^{(-1/slope)}$ and % efficiency = 100 · (PCR efficiency - 1) (see Table S4). Primer specificities were verified by melting curve analysis (Figure S3).

Puromycin incorporation assay

Strain SF370 was grown to mid-logarithmic growth phase, exposed to 1 mM H_2O_2 or water as a control for 20 min, and treated with 10 µg/mL puromycin (Sigma-Aldrich) for further 10 min. Proteins were isolated by bead-beating and quantified using Bio-Rad Protein Assay. Equal protein amounts were separated on Any kD Mini-PROTEAN TGX Precast Protein Gels (Bio-Rad), transferred onto 0.45 µm PROTRAN nitrocellulose membranes in 1x Towbin buffer with 20% methanol and stained with 0.2% Ponceau S in 3% acetic acid as a loading control. After destaining, membranes were blocked in 5% skim milk in 1x TBS with 0.1% Tween-20, incubated first with α-puromycin (1:2,000; MABE342, Sigma-Aldrich) and second with HRP-linked ECL Mouse IgG (1:5,000; GE Healthcare), and developed using SuperSignal West Pico Chemiluminescent Substrate (Thermo Scientific).

Identification of A-to-I editing events by RNA sequencing

We developed a pipeline to identify A-to-I editing sites based on a recent publication (10). Since libraries of genomic DNA did not contain Unique Molecular Identifiers (UMIs), UMI extraction was performed using UMI Tools (v1.0.1) for RNA samples only. Adapter sequences were removed using Cutadapt (v2.10) (11), and reads were then mapped to the respective

reference genome (NC_002737.2 for *S. pyogenes* SF370, NZ_CP008776.1 for *S. pyogenes* 5448) and the PhiX genome using BWA-MEM (v.0.7.17) (12). We sorted and indexed the obtained BAM files using Samtools (v1.9) (13). UMI-based PCR deduplication was performed using UMI Tools (v1.0.1) and reference demultiplexing was performed using Samtools. For paired-end reads, overlapping reads were merged and read duplication artefacts created by the mapping algorithm were removed. We masked homopolymer stretches longer than 4 bp from the reference genome and identified single nucleotide polymorphisms (SNPs). Specifically, we extracted and filtered unique reads for each genomic position based on site-specific nucleotides with a Phred quality score of at least 30 and a minimum distance of at least 4 nt from the end of the reads. We further filtered sites with a minimum coverage of 20, at least two supporting reads per strand direction, and a minimum frequency of 0.01. In RNA datasets, we filtered SNPs for the observation of only two different nucleotides, with one being the reference genome. We removed SNPs in the RNA and DNA datasets positioned at masked sites. Next, we compared the SNPs and their positions in the RNA with the DNA dataset and removed them if they were also observed in the DNA dataset. For the H_2O_2 stress experiment, we identified SNPs by comparing only RNA sequences to previously sequenced genomic DNA from strain SF370 (from the dataset for initial identification of editing sites across growth phases). A site was further considered if it was observed in two independent replicates. We then filtered for A-to-G transitions and reported them.

We manually curated reported A-to-I editing sites. To improve comparability with the publication on A-to-I editing in *E. coli* (10) and to retrieve high-confidence sites, we additionally filtered positions for a minimum frequency of 3% in two independent replicates. We excluded sites in ribosomal RNA to avoid biases due to rRNA depletion and because of the presence of known modifications (*e.g.*, m^6A1518/1519 in 16S rRNA). We found false-positive hits for position 22 in transfer RNAs. Due to N1 methylation of adenosine-22, guanosine was preferentially introduced during reverse transcription in the context of the corresponding local sequence (14), so we excluded sites at tRNA position 22. We checked editing positions in intergenic regions (IGRs) against the orientation of the transcript's coding sequence (CDS) and removed inconsistent sites. We also excluded identified sites adjacent to the 3′ end of tRNA genes due to the post-transcriptional addition of the CCA terminus. In case of position 450,537 in the 5′ UTR of *SPy_0558*, RT-PCR revealed the absence of a portion of

the 5′ UTR in RNA but not gDNA samples, leading to misaligned reads and false-positive site identification (data not shown). Lastly, we manually checked sites for mapping artefacts or unusual processing events using the Integrative Genomics Viewer (15). We confirmed by BLAST analysis that the editing sites identified were not in misalignment-prone paralogous sequences resulting from gene duplication events (31 bp window around editing position; data not shown).

We identified two positions located in poorly annotated regions of the SF370 reference genome. First, position 819,206 is annotated in the CDS of *speI* (*SPy_1007*) at codon position 9, but according to previous reports – given the signal peptide sequence and translation initiation at an alternative GTG start codon – the position lies within codon 43 (updated *speI* annotation: NC_002737.2:819,078..819,857) (16). Second, position 962,967 is located upstream of the annotated *gid* CDS (*SPy_1173*), but analysis of *gid* annotations in different strains suggests translation initiation from an alternative GTG start codon further upstream, and the identified editing position is thus located within codon 6 (updated *gid* annotation: NC_002737.2:962,952..964,346).

SUPPLEMENTARY FIGURES

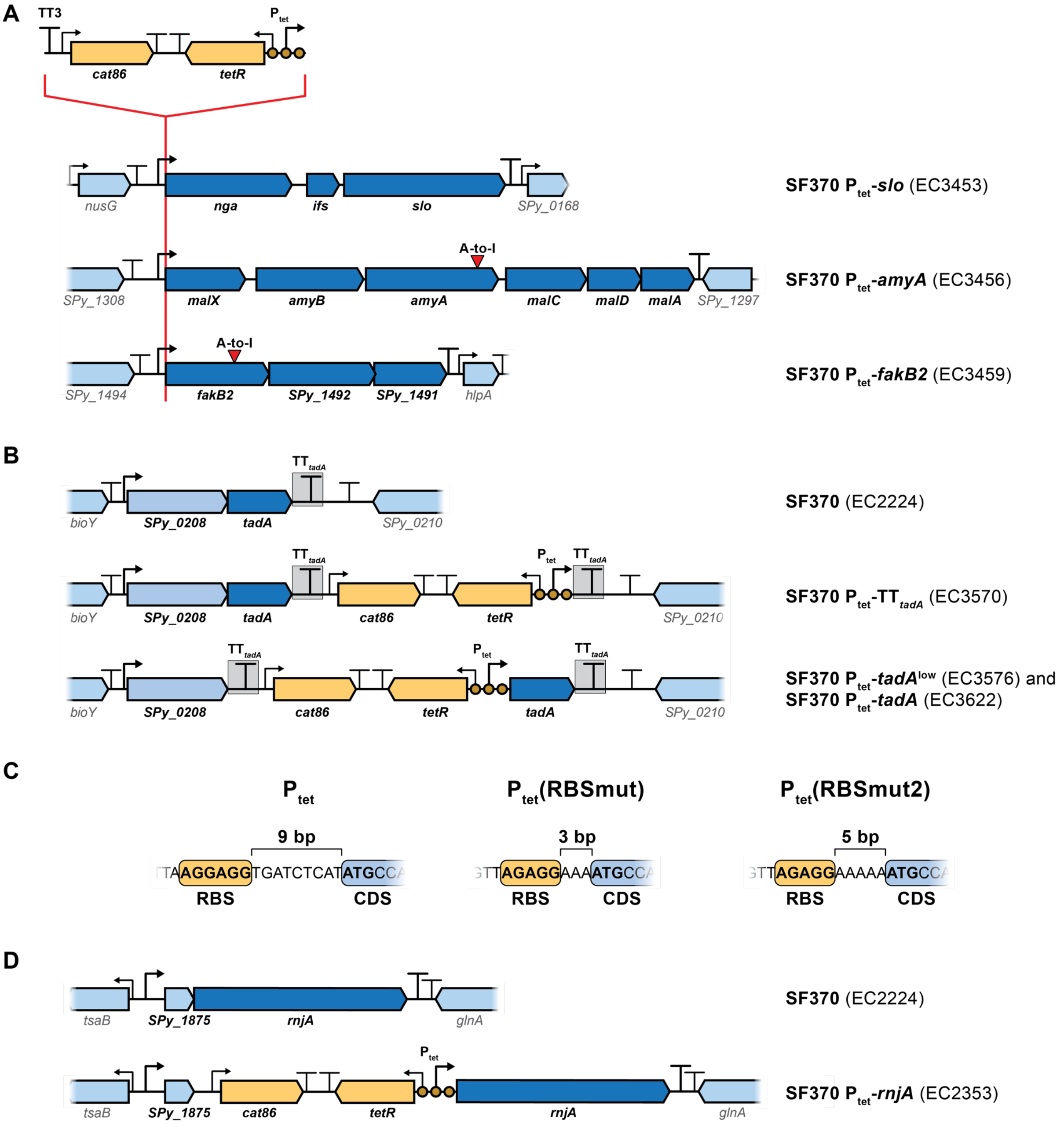

Figure S1. Schematic maps of gene loci for P_{tet}-harboring strains. (A) To test the influence of gene expression on A-to-I editing levels, the inducible P_{tet} promoter cassette was inserted at the transcriptional start site of two selected editing target operons (*malX-amyBA-malCDA* for strain EC3456 and *fakB2-SPy_1492-SPy_1491* for strain EC3459) and a control operon (*nga-ifs-slo* for strain EC3453). Genes of the affected operons are shown in dark blue, while the surrounding genes are shown in light blue. A-to-I editing sites are marked by red arrows. The

P_{tet} cassette including the *cat86* resistance marker is shown in yellow. Promoters and terminators are indicated. (B) The wildtype locus of the bicistronic *SPy_0208-tadA* operon in strain SF370 (EC2224) is shown in the upper panel with *tadA* highlighted in dark blue and the *tadA* terminator (TT_{tadA}) framed by a grey box. For the control strain EC3570, the P_{tet} promoter cassette was inserted downstream of TT_{tadA}, and TT_{tadA} was additionally used as terminator directly downstream of the P_{tet} promoter (middle panel). For the AHT-inducible *tadA* strains EC3576 and EC3622, the P_{tet} cassette was inserted between *SPy_0208* and *tadA*, and TT_{tadA} was additionally used as terminator for *SPy_0208* (lower panel). For P_{tet}-*tadA*^{low} (EC3576), P_{tet}(RBSmut) was used, whereas P_{tet}-*tadA* (EC3622) employs P_{tet}(RBSmut2) as depicted in (C). (C) Different ribosome binding site (RBS) variants used together with the P_{tet} promoter are shown with the RBS highlighted in yellow and the coding sequence in blue. The distance between RBS and start codon (in bold) is depicted. (D) The wildtype locus of the bicistronic *SPy_1875-rnjA* operon in strain SF370 (EC2224) is shown in the upper panel with *rnjA* highlighted in dark blue. For the inducible P_{tet}-*rnjA* strain EC2353, the P_{tet} cassette was inserted between *SPy_1875* and *rnjA* as previously reported (2).

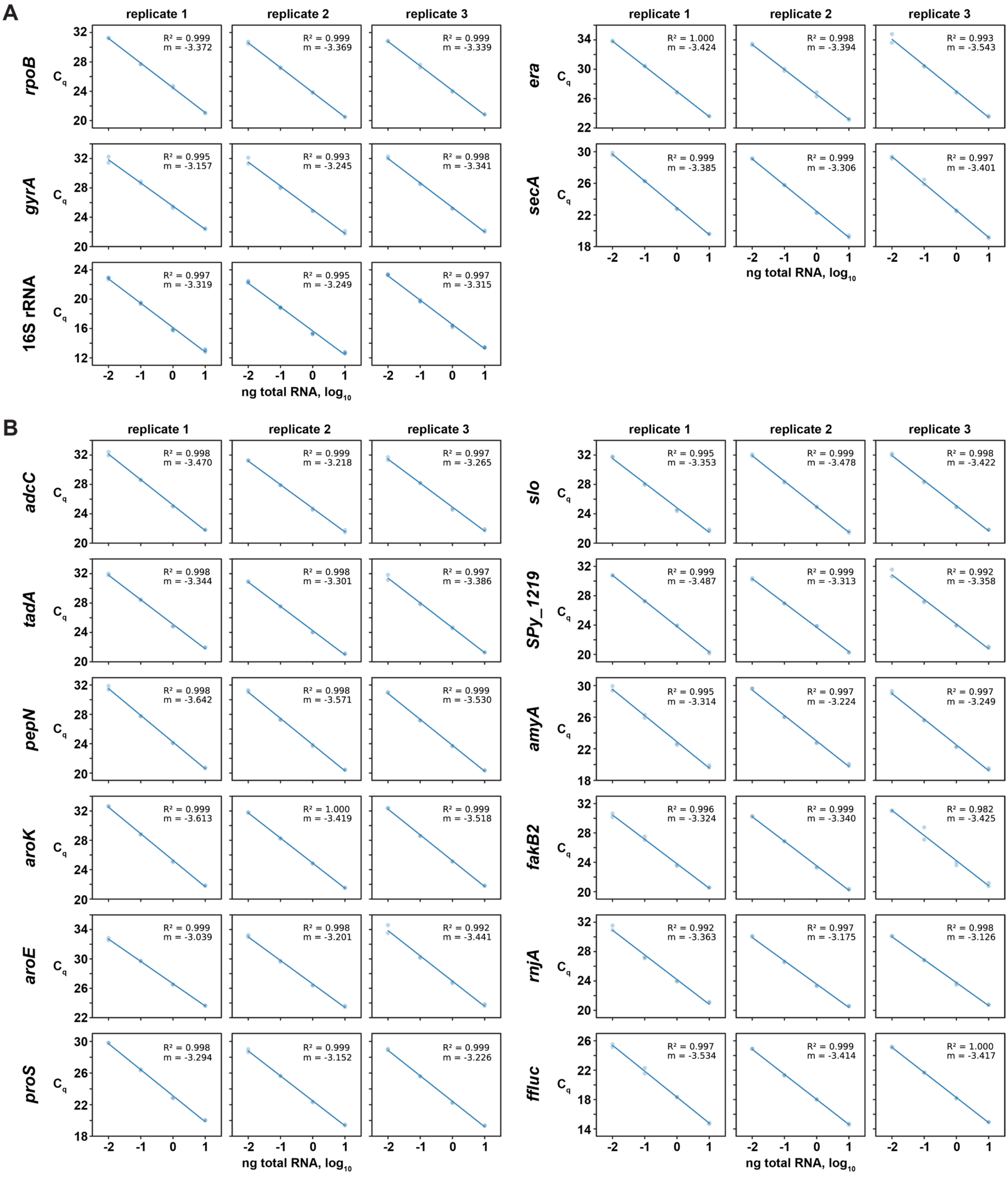

Figure S2. Assessment of qRT-PCR primer efficiencies. qRT-PCR was performed as described in Supplementary Methods with a $\log_{10}$ dilution series of RNA input (10 ng, 1 ng, 0.1 ng and 0.01 ng RNA per 20 µL reaction). DNase-treated total RNA from strain SF370 carrying the *ffluc*-expressing pEC3045 (mid-logarithmic growth phase, 0 ng/mL erythromycin) was used for all experiments except for target gene *slo* for which RNA from strain EC3453 (mid-logarithmic growth phase, 100 ng/mL AHT) was used. Experiments were performed in triplicate with technical duplicates each. C_q values were plotted against the $\log_{10}$-transformed RNA input, and

coefficient of determination (R^2) and slope (m) of the regression line are shown for each replicate. Results for reference genes are shown in (A) and for target genes in (B).

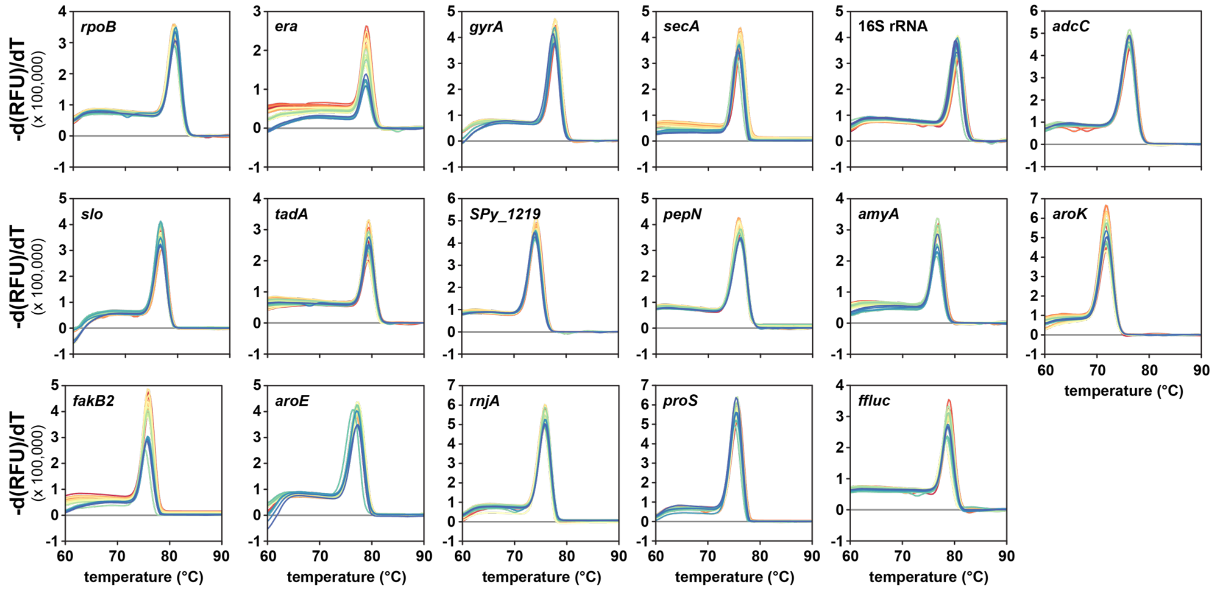

Figure S3. Assessment of qRT-PCR primer specificity. qRT-PCR was performed as described for Figure S2, and melting curves were recorded for all target gene and each individual well (color-coded). The temperature-dependent change in fluorescence (-d(RFU)/dT) was plotted against the respective temperature. Single peaks indicate single amplicons without contaminating by-products.

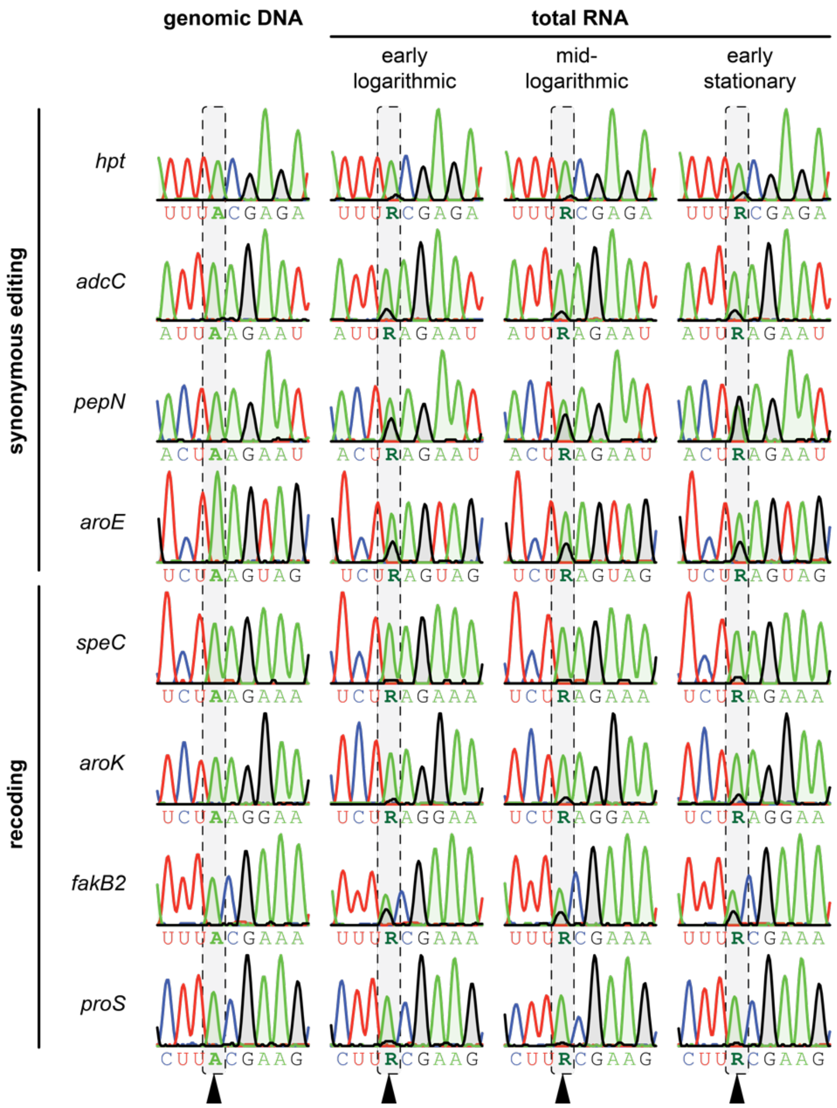

Figure S4. Validation of selected A-to-I editing sites in *S. pyogenes* by Sanger sequencing. Total RNA was extracted from early logarithmic, mid-logarithmic and early stationary growth phase. Selected A-to-I editing sites identified by NGS were validated by Sanger sequencing in biologically independent samples. Chromatograms of four selected synonymous editing events are shown in the upper panels and those of four recoding events in the lower panels. Chromatograms for amplicons derived from genomic DNA of SF370 are shown as controls (left). The corresponding editing positions are indicated with black arrows. Nucleotide sequences are shown below each chromatogram. Sequencing traces of adenosine and guanosine are shaded in green and grey, respectively, for better visualisation.

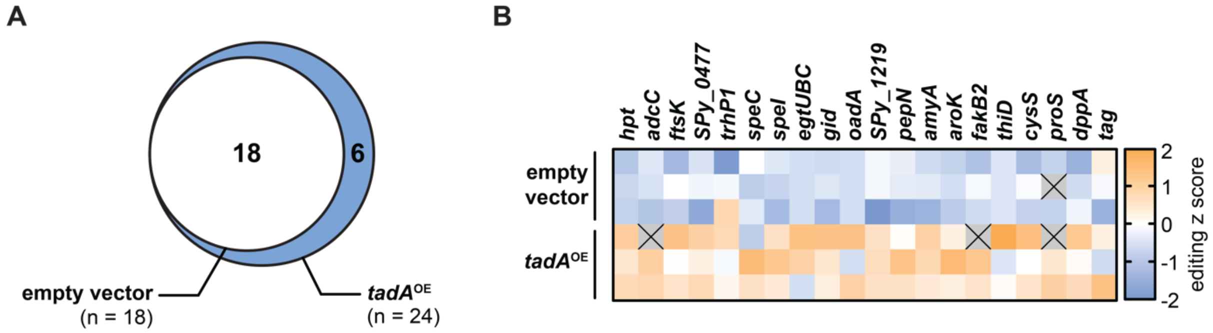

Figure S5. Validation of A-to-I editing sites by *tadA* overexpression. A *tadA* overexpression plasmid (pEC2813) and the empty vector control (pEC2812) were introduced by transformation into *S. pyogenes* SF370, and cultures were grown to mid-logarithmic phase in C medium in the presence of kanamycin. Total RNA was extracted and NGS was performed to examine editing levels. (A) Venn diagram of the identified editing sites in the empty vector control and upon overexpression of *tadA*. (B) Editing z scores for each replicate are shown in the heatmap. For replicates depicted as grey tiles with a black cross, editing was not determined due to filtering steps.

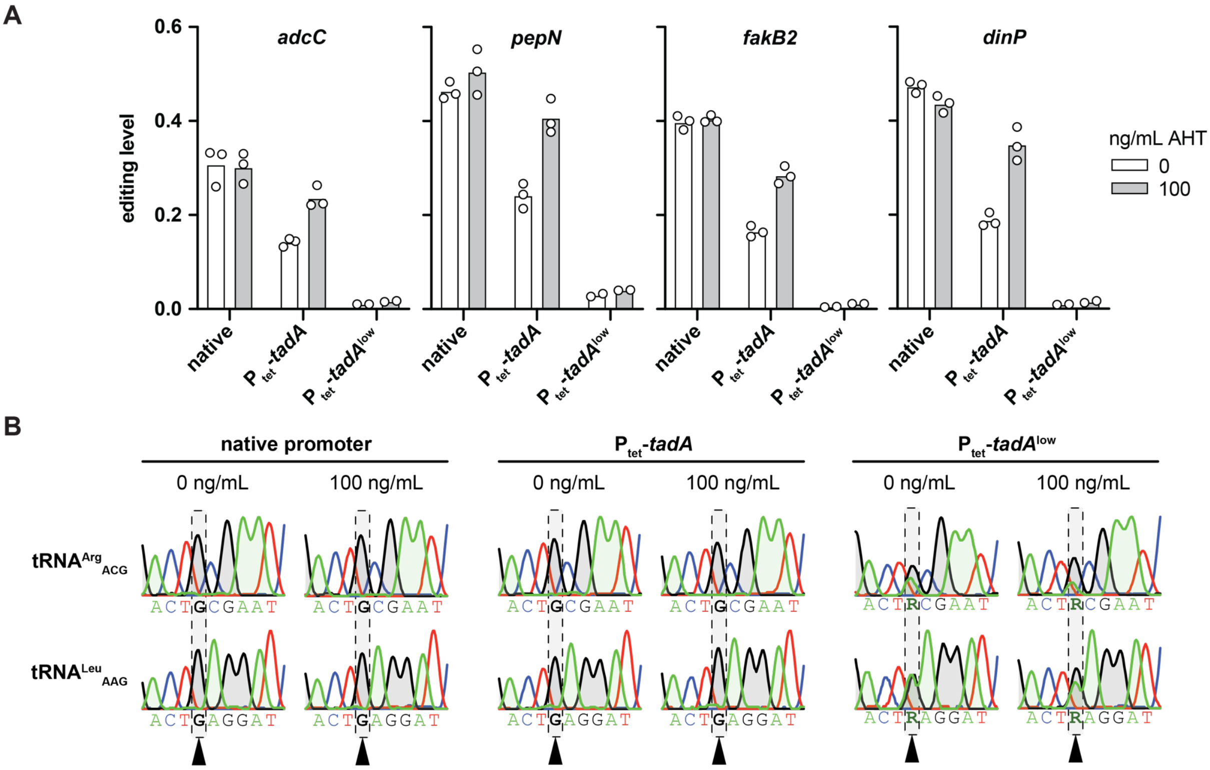

Figure S6. A-to-I editing upon conditional expression of *tadA* using P_{tet}. As control for native expression regulation, the P_{tet} cassette was inserted downstream of *tadA* ("native", strain EC3570). Two different mutant versions of the P_{tet} ribosome binding site (RBS) with different spacing between RBS and start codon were used when inserting the P_{tet} cassette upstream of *tadA* (compare Figure S1C; P_{tet}-*tadA* (EC3622) and P_{tet}-*tadA*^{low} (strain EC3576), respectively). Cultures were grown to mid-logarithmic phase in C medium, split in two cultures and treated with or without 100 ng/mL AHT for 30 min, before total RNA was extracted. Editing levels were determined by Sanger sequencing. Experiments were performed in triplicates for EC3570 and EC3622 and in duplicates for EC3576. (A) mRNA editing. Editing levels of *adcC*, *pepN*, *fakB2* and *dinP* were consistently reduced under non-induced conditions in P_{tet}-*tadA*, but reverted back close to native editing levels in the presence of 100 ng/mL AHT. In P_{tet}-*tadA*^{low}, however, editing is severely reduced to less than 5% for all tested genes even in the presence of AHT. (B) tRNA editing. Both $tRNA^{Arg}_{ACG}$ and $tRNA^{Leu}_{AAG}$ are nearly completely modified during native *tadA* expression and in P_{tet}-*tadA*, irrespective of the presence of AHT. Only in P_{tet}-*tadA*^{low}, both tRNAs are no longer fully modified to I34, and addition of AHT only partially restores tRNA editing. Exemplary chromatograms are shown with the editing position indicated with black arrows. Nucleotide sequences are shown below each chromatogram. Sequencing traces of adenosine and guanosine are shaded in green and grey, respectively, for better visualisation.

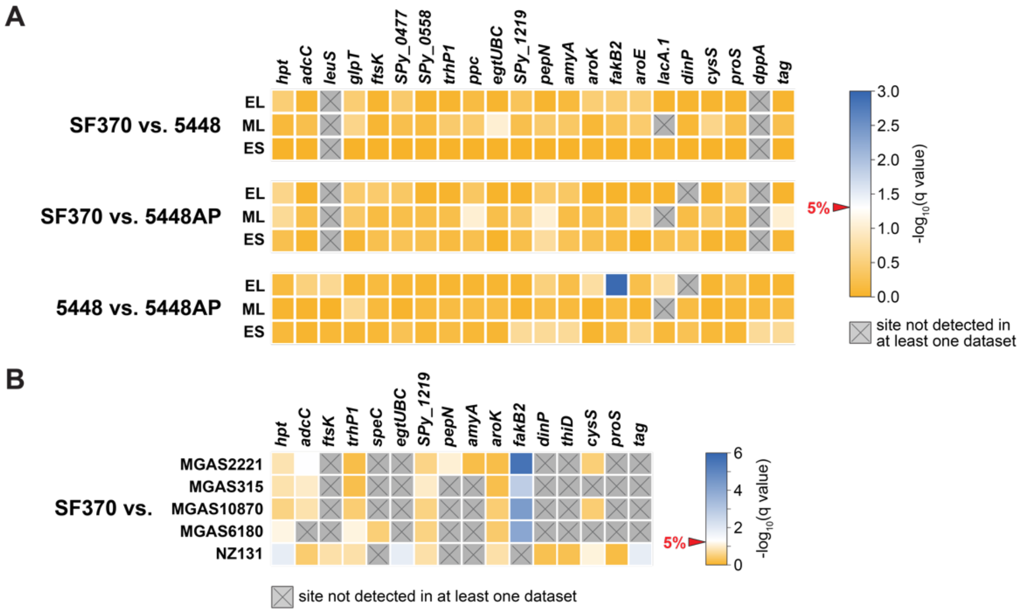

C

	synonymous editing								recoding																
	hpt	*adcC*	*SPy_0558*	*trhP1*	*ppc*	*SPy_1219*	*pepN*	*aroE*	*glpT*	*ftsK*	*SPy_0477*	*pplD*	*egtUBC*	*amyA*	*aroK*	*fakB2*	*arcD*	*lacA.1*	*dinP*	*thiD*	*cysS*	*proS*	*pflA*	*tag*
Lla	94	47	0	47	0	32	46	49	30	46	70	47	45	0	47	0	0	23	47	46	47	47	0	0
Sag	50	51	0	51	51	53	51	51	49	51	51	42	51	0	51	51	0	58	51	51	51	51	51	51
San	7	7	0	7	7	0	7	7	0	7	7	7	3	0	7	7	7	1	7	7	7	7	7	6
Sdy	21	21	0	21	21	21	20	21	15	21	8	21	21	0	21	21	21	31	21	21	21	21	23	22
Seq	15	15	0	15	15	0	14	15	0	13	15	15	15	15	15	14	15	15	15	15	15	15	15	15
Sin	6	6	0	6	6	2	6	6	6	6	6	6	6	6	6	6	6	0	6	6	6	6	6	6
Smi	5	5	0	5	5	0	5	5	0	5	2	0	5	0	5	5	0	5	5	5	5	5	0	5
Smu	15	15	0	15	14	1	15	15	0	15	15	15	15	0	15	14	0	15	15	15	15	16	15	15
Spn	53	53	0	52	53	0	53	53	0	53	0	0	53	0	53	53	52	53	53	53	53	53	53	53
Spy	114	114	49	115	115	115	115	211	115	115	114	115	115	41	115	115	114	230	115	115	115	115	114	115
Ssa	11	11	0	11	11	0	11	11	0	11	0	11	11	0	11	11	0	0	11	12	11	11	0	12
Ssu	34	34	0	34	34	34	0	34	0	34	0	34	33	3	35	34	33	37	34	50	33	34	32	34
Sth	35	35	0	35	35	0	35	35	0	34	0	35	0	0	35	35	0	0	35	36	35	36	0	35

Figure S7. Conservation of A-to-I editing sites in *S. pyogenes* and *Streptococcaceae* members. (A) Pairwise comparison of editing levels in strains SF370, 5448 and 5448AP (Tables S6 and S8). Statistical analysis was performed using t-tests, and *P*-values were adjusted using the procedure of Benjamini, Krieger and Yekutieli. Log_{10}-transformed q values are shown for each gene and growth phase in a heatmap. (B) Pairwise comparison of editing levels as described for (A) in publicly available RNA-seq data (as in Figure 3B) and strain SF370 (matched media at mid-logarithmic phase: C medium as in Table S7, THY as in Table S10). (C) The number of identified homologs of *S. pyogenes* SF370 editing target genes in other *Streptococcaceae* species is shown. Species: *Lactococcus lactis* (*Lla*), *Streptococcus agalactiae* (*Sag*), *S. anginosus* (*San*), *S. dysgalactiae* (*Sdy*), *S. equi* (*Seq*), *S. iniae* (*Sin*), *S. mitis* (*Smi*), *S. mutans* (*Smu*), *S. pneumoniae* (*Spn*), *S. pyogenes* (*Spy*), *S. salivarius* (*Ssa*), *S. suis* (*Ssu*), and *S. thermophilus* (*Sth*).

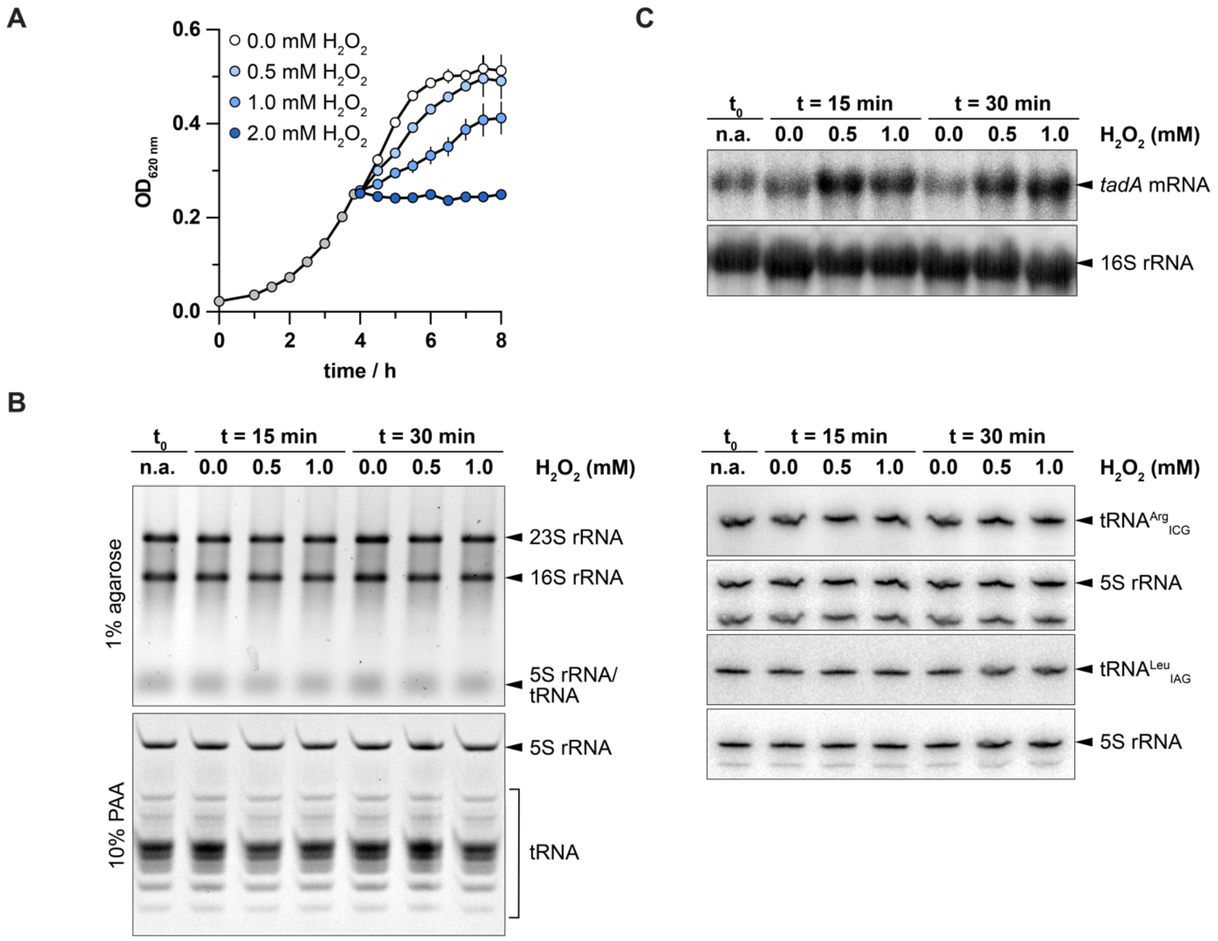

Figure S8. Cellular response of *S. pyogenes* SF370 to H_2O_2 exposure. (A) *S. pyogenes* was grown to mid-logarithmic growth phase in C medium (grey circles) and challenged with different concentrations of H_2O_2 (0.5, 1.0 and 2.0 mM; circles in shades of blue) or H_2O as control (0.0 mM H_2O_2, white circles). OD_{620} was measured every 30 min to follow growth. (B) Culture aliquots were taken before and after challenge with H_2O_2 for the indicated time and concentration, and total RNA was extracted, separated by denaturing agarose (top left panel) or polyacrylamide gel electrophoresis (bottom left panel) and stained using SYBR™ Gold Nucleic Acid Gel Stain. In addition, the abundances of the two TadA target tRNAs were determined by denaturing polyacrylamide gel electrophoresis and consecutive Northern blotting using 5S rRNA as a loading control (right panel). (C) *tadA* mRNA abundance after H_2O_2 exposure was determined by denaturing agarose gel electrophoresis and subsequent Northern blotting using 16S rRNA as a loading control. rRNA, tRNA and mRNA are labelled on the right. Experiments were performed in biological triplicates. n.a. (not applicable)

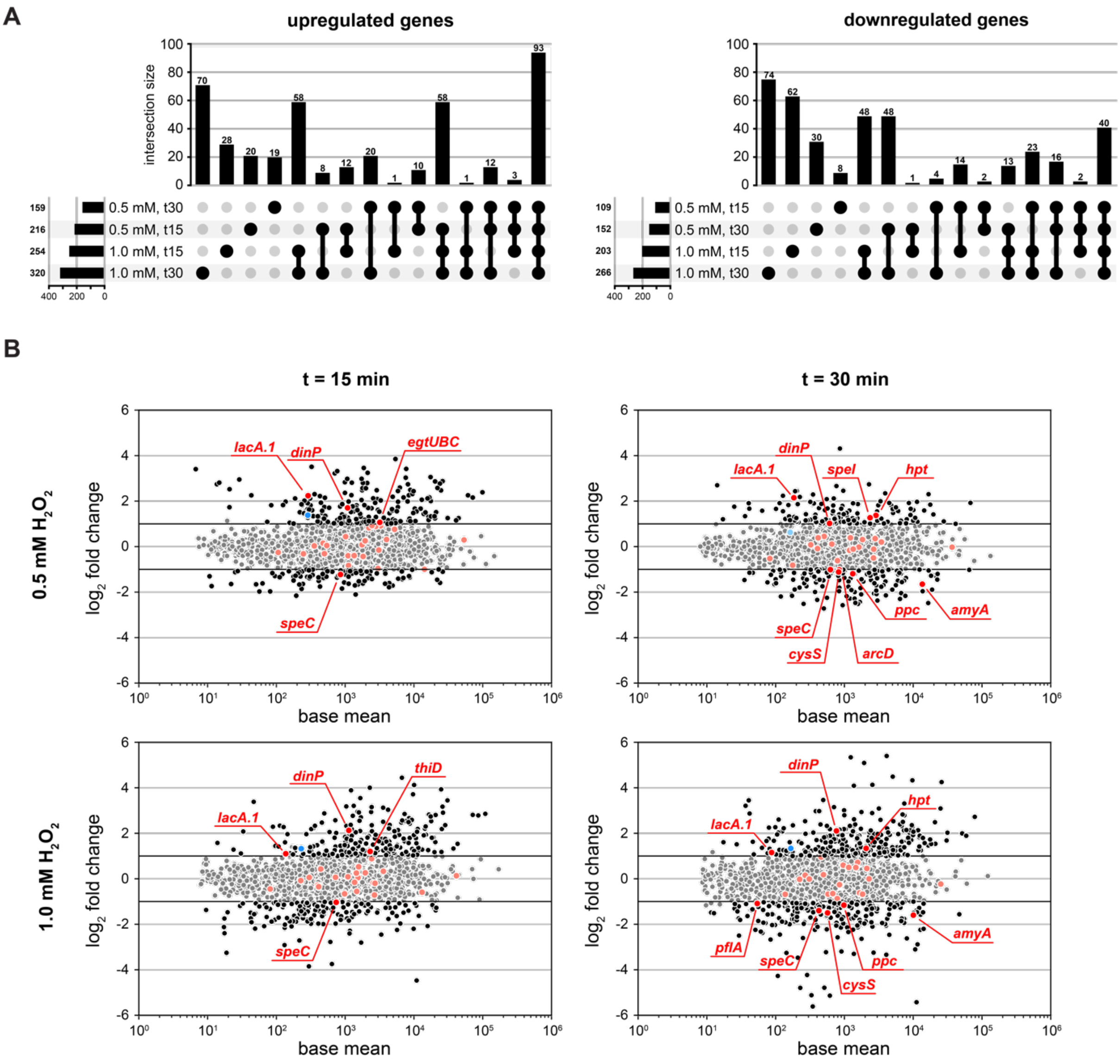

Figure S9. Differential expression analysis of *S. pyogenes* SF370 exposed to H_2O_2. Differential expression analysis was performed relative to the untreated control at the respective time point with a minimum $\log_2$ fold change of 1 or -1 and an adjusted p-value no more than 0.05. (A) The overlaps of differentially expressed genes (including small RNAs) upon challenge of *S. pyogenes* SF370 with different concentrations of H_2O_2 for 15 min or 30 min are illustrated by UpSet plots (left: upregulated genes; right: downregulated genes). (B) MA plots are shown for each stress condition with editing target genes (as in Table 1) highlighted in red and *tadA* in blue. Pale circles indicate a non-significant change in expression. Small RNAs were omitted for better visualisation.

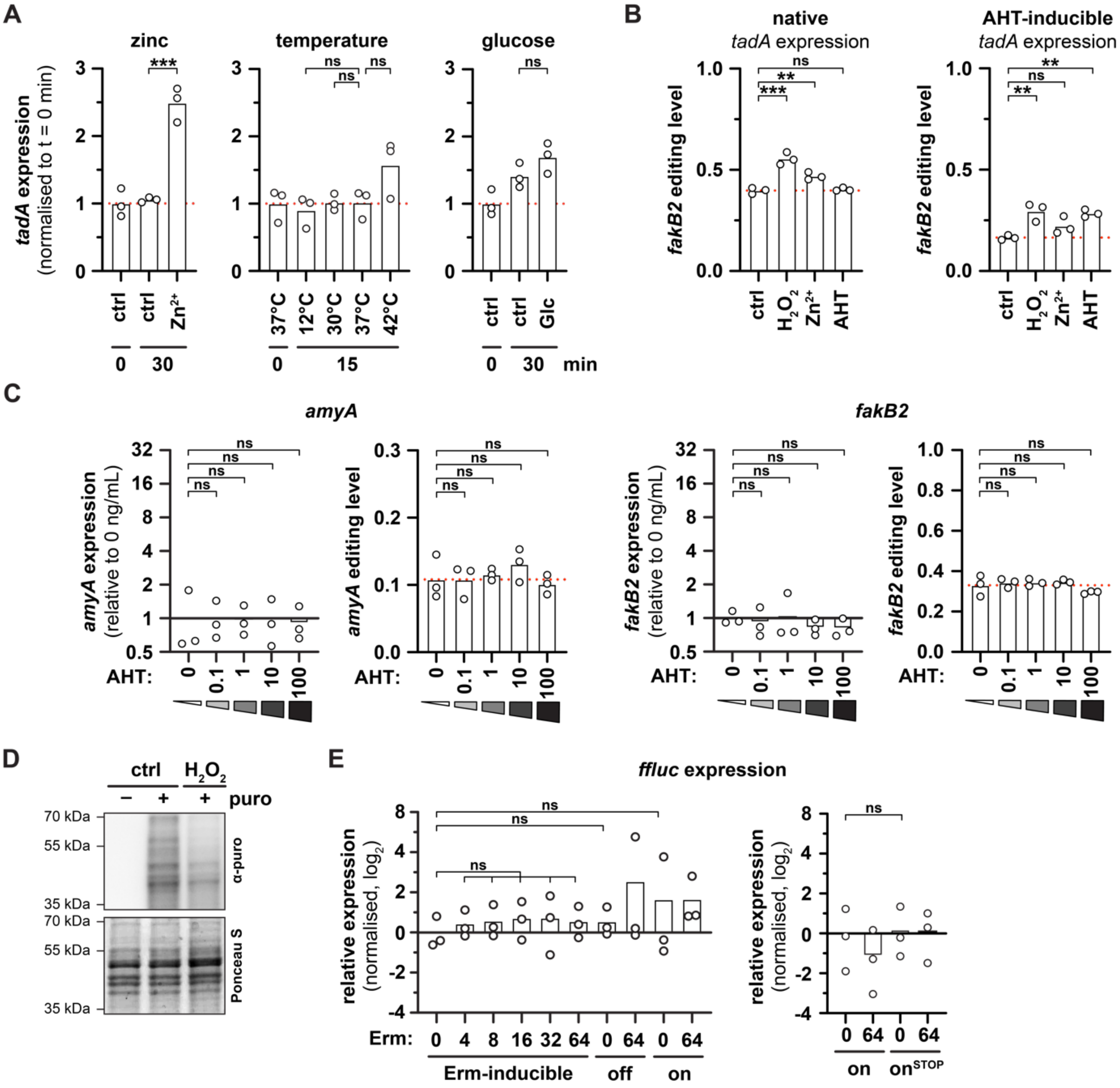

Figure S10. The dynamics of stress-dependent mRNA editing is not governed by *tadA* expression, mRNA expression or mRNA translation. (A) Effect of different stimuli on *tadA* expression. *S. pyogenes* SF370 was grown to mid-logarithmic growth phase and exposed for the indicated time to different stimuli. *tadA* expression was then examined by qRT-PCR with *gyrA*, *rpoB* and *era* as reference genes and normalised to the mean expression at t = 0 min. Cells were exposed to 0.5 mM $ZnSO_4$ (left), different temperatures (middle) or 0.5% (w/v) glucose (right). Untreated cultures served as control ("ctrl"). Statistical analysis was performed using one-way ANOVA and Dunnett's post-hoc test (temperature) or unpaired t test (zinc and glucose). (B) Editing of *fakB2* in response to H_2O_2, zinc and AHT exposure in the native (left)

and AHT-inducible (right) *tadA* expression strains (compare Figure 5C-D). (C) Effect of AHT on mRNA abundance and mRNA editing of *amyA* (left panels) and *fakB2* (right panels). The AHT-inducible P_{tet} promoter cassette was integrated upstream of the *nga-ifs-slo* operon as control, and the strain was grown and analysed as described for Figure 6A. (D) Changes in translation rate in response to H_2O_2. *S. pyogenes* was exposed at mid-logarithmic phase to 1.0 mM H_2O_2 for 20 min and treated with puromycin ('puro') for a further 10 min. The rate of translation was examined by Western blotting against puromycin. Results from one exemplary replicate are shown. Statistical analysis was performed using one-way ANOVA and Dunnett's post-hoc test. (E) *ffluc* expression in *ermB* regulatory region-dependent luminescence reporter assay. Strains were grown and treated as described in Figure 6B, and *ffluc* expression was examined by qRT-PCR with *gyrA* and *rpoB* as reference genes, normalised to the mean expression at 0 ng/mL erythromycin (Erm) in the wildtype reporter and $\log_2$ transformed. (A-E) Statistical analysis was performed as described in the main figure, unless otherwise stated. *** $P < 0.001$; ** $P < 0.01$; * $P < 0.05$; ns, not significant.

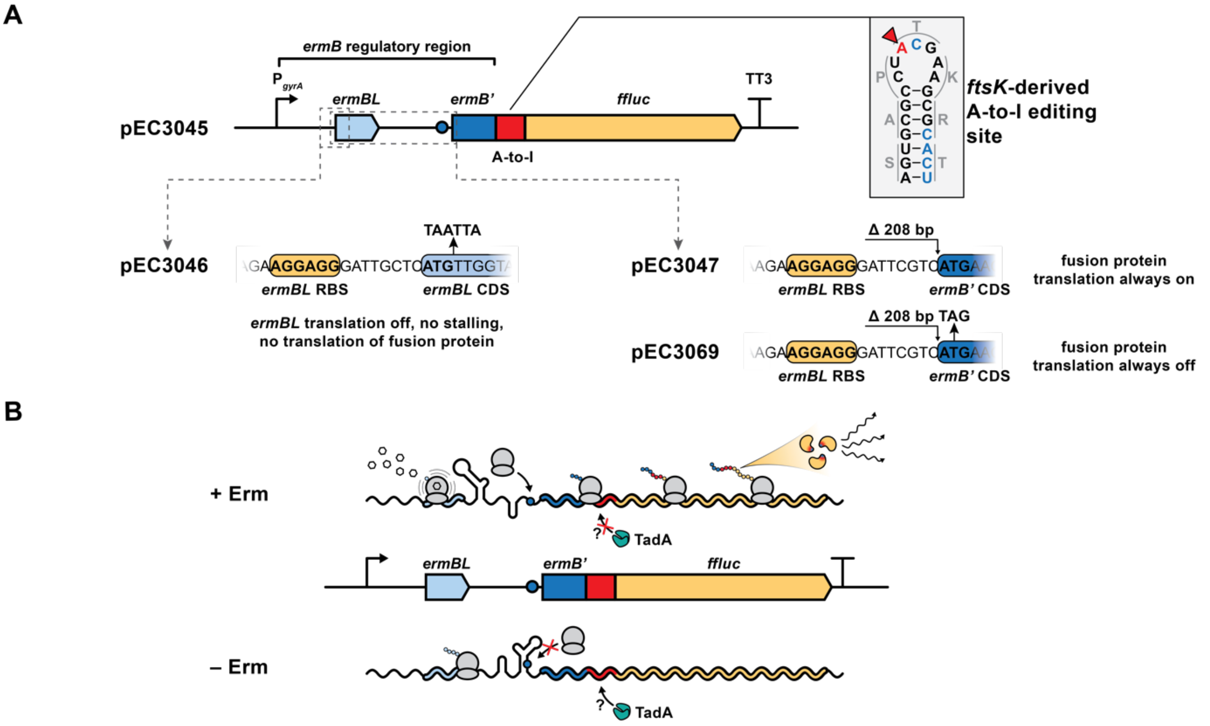

Figure S11. Schematic overview of the *ermBL*-based reporter assay. (A) In the wildtype reporter plasmid pEC3045, the constitutive promoter P_{gyrA} drives the transcription of the reporter mRNA, consisting of the *ermB* regulatory region (including the leader peptide *ermBL*

in light blue and the first 30 nt of the *ermB* coding sequence in dark blue) with the *ermB* fragment fused in-frame to an *fstK*-like A-to-I editing site (in red) and the firefly luciferase *ffluc* (in orange). The *ftsK*-like editing site was carefully adjusted (blue nucleotides) to strengthen the secondary structure and achieve high editing levels (at the adenosine highlighted in red) (right side). Two mutant versions were designed to (i) shut down translation irrespective of the presence of erythromycin (pEC3046; mutation of start codon of *ermBL*) or to (ii) allow constitutive translation of the fusion protein (pEC3047; deletion of 208 bp comprising *ermBL* and the *ermBL*-*ermB*' intergenic region). As an additional control for pEC3047, the start codon of the constitutively translated fusion protein was mutated (pEC3069). (B) For the wildtype reporter, translation of the fusion protein is inhibited in the absence of erythromycin (– Erm) and the editing site is assumed to be accessible to TadA (in turquoise). In contrast, the presence of erythromycin (+ Erm) results in ribosome stalling in the leader peptide *ermBL* and increased translation of the fusion protein, thereby possibly masking the editing site from TadA.

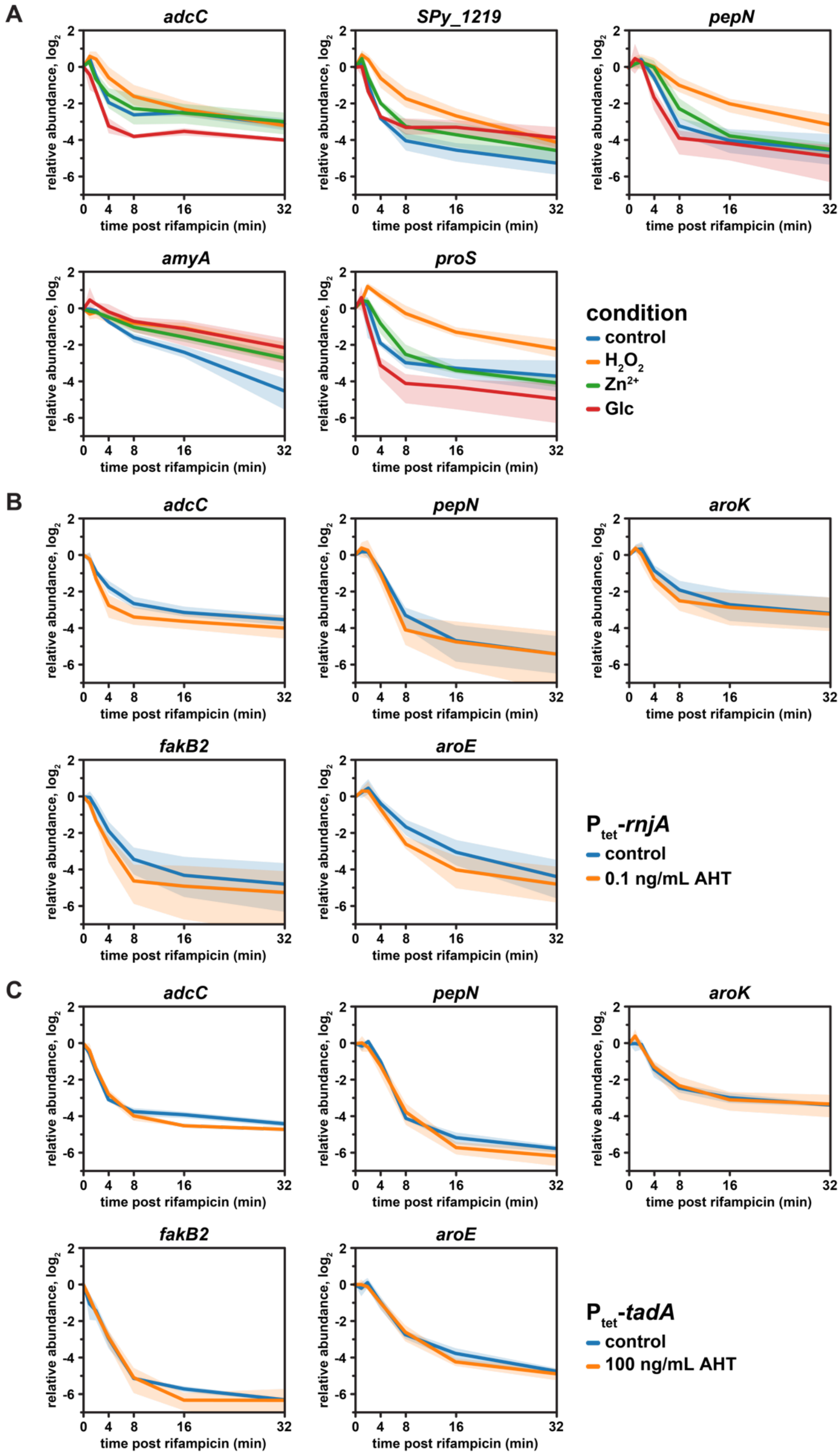

Figure S12. Changes in mRNA turnover for selected editing target genes. (A) *S. pyogenes* SF370 was grown to mid-logarithmic phase and exposed to 1.0 mM H_2O_2, 0.5 mM $ZnSO_4$ or

0.5% glucose for 30 min. (B, C) *S. pyogenes* P_{tet}-*rnjA* (B) and P_{tet}-*tadA* (C) were grown in the presence or absence of AHT to mid-logarithmic growth phase. (A – C) Rifampicin was added at 250 µg/mL and samples were taken right before and after 1 min, 2 min, 4 min, 8 min, 16 min, and 32 min. mRNA abundances were measured by qRT-PCR, normalised first to 16S rRNA levels and second to t = 0 min for each gene. Mean mRNA abundances are shown for each gene as a function of time after rifampicin addition, with standard deviation represented by shaded areas.

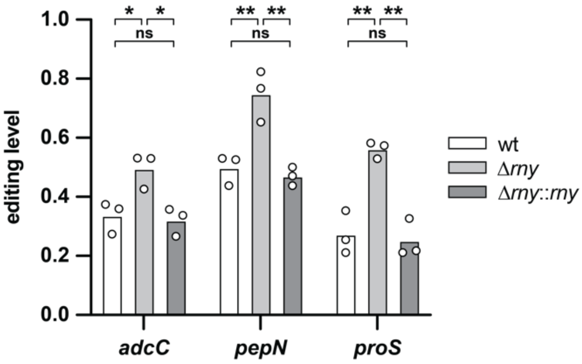

Figure S13. Effect of *rny* deletion on A-to-I editing. *S. pyogenes* SF370 wild type, Δ*rny* and Δ*rny*::*rny* were grown to early stationary phase in THY. A-to-I editing levels were analysed by Sanger sequencing for *pepN* and two target genes known to be encoded in RNase Y-processed operons (*adcC* and *proS*) (17). Statistical analysis was performed using one-way ANOVA and Tukey's post-hoc test. ** $P < 0.01$; * $P < 0.05$; ns, not significant.

SUPPLEMENTARY TABLES

Table S1. Strains used in this study.

Strain name	Strain code	Relevant characteristics	Source
Streptococcus pyogenes			
**Wild types**			
SF370	EC2224	M1 serotype	ATCC 700294
5448	EC2615	M1T1 serotype	(18)
5448AP	EC2617	M1T1 serotype, *covS*	(18)
**Isogenic mutants**			
SF370 Δ*rny*	EC2246	SF370 Δ*rny*::lox72	(3)
SF370 Δ*rny*::*rny*	EC2298	EC2246 Δlox72::*rny*-TT3-lox72	(19)
SF370 P_{tet}-*rnjA*	EC2353	SF370 *SPy_1875*::*cat86*-P_{tet}*	This study
SF370 P_{tet}-*slo*	EC3453	SF370 P_{nga}::TT3-*cat86*-P_{tet}	This study
SF370 P_{tet}-*amyA*	EC3456	SF370 P_{malX}::TT3-*cat86*-P_{tet}	This study
SF370 P_{tet}-*fakB2*	EC3459	SF370 P_{fakB2}::TT3-*cat86*-P_{tet}	This study
SF370 P_{tet}-TT_{tadA}	EC3570	SF370 TT_{tadA}::*cat86*-P_{tet}(RBSmut)-TT_{tadA}	This study
SF370 P_{tet}-*tadA*^{low}	EC3576	SF370 *SPy_0208*::TT_{tadA}-*cat86*-P_{tet}(RBSmut)	This study
SF370 P_{tet}-*tadA*	EC3622	SF370 *SPy_0208*::TT_{tadA}-*cat86*-P_{tet}(RBSmut2)	This study
Escherichia coli			
TOP10		Host for cloning	Invitrogen
NEB 5-alpha		Host for cloning	NEB
NiCo21(DE3)		Host for protein production	NEB

Promoters are denoted as P with the related gene name. The AHT-inducible P_{tet} cassette harbours the repressor *tetR* and two divergent promoters with three operator sites, and is separated from the chloramphenicol resistance gene *cat86* by two T4 terminators as previously described (1, 2). A mutant version of P_{tet} with *tetR*(A582C) is shown as P_{tet}*. Mutations of the ribosomal binding site as shown in Figure S1 are described as P_{tet}(RBSmut) and P_{tet}(RBSmut2). Transcriptional terminators are denoted as TT, with TT3 as the phage T3 terminator.

Table S2. Oligonucleotides used in this study.

**Purpose**	**Code**	**Sequence 5′-3′**[a]	**F/R**[b]	**Usage**[c]
**Sanger sequencing of A34-to-I34 editing of transfer RNAs**				
E. coli argQ / tRNA$^{Arg}_{ACG}$	OLEC9821	*GCATCCGTAGCTCAGCTGG*	F	RT-PCR
	OLEC9822	*TGGTGCATCCGGGAGGATTC*	R	RT
	OLEC9823	TAATACGACTCACTATAGGG*TGGTGCATCCGGGAGGATTC*	R	RT-PCR
SPy_t37 / tRNA$^{Arg}_{ACG}$	OLEC8776	*GCACCCTTAGCTCAACTGG*	F	RT-PCR
	OLEC8777	*TGGTGCACCCTAGAGGAG*	R	RT
	OLEC9171	TAATACGACTCACTATAGGG*TGGTGCACCCTAGAGGAG*	R	RT-PCR
*SPy*_t52 / tRNA$^{Leu}_{AAG}$	OLEC8778	*GCGGGGATGGCGGAATTG*	F	RT-PCR
	OLEC8779	*TGGTGCGGAGAGTGGGAC*	R	RT
	OLEC9172	TAATACGACTCACTATAGGG*TGGTGCGGAGAGTGGGAC*	R	RT-PCR
all tRNAs	OLEC1889	*TAATACGACTCACTATAGGG*	F	SEQ
**Sanger sequencing of A-to-I editing of messenger RNAs**				
SPy_0014 / hpt	OLEC10617	*CATGGCGGTACATCTAGCAG*	F	RT-PCR, SEQ
	OLEC10618	*CGTAATCTAAACCAAAGCCTACG*	R	RT-PCR
SPy_0093 / adcC	OLEC11128	*GAATTTGTCACCATGACCGGTG*	F	RT-PCR
	OLEC11129	*GCTTGCACATGCTCTTCATCG*	R	RT-PCR, SEQ
SPy_0711 / speC	OLEC10605	*CAGTCATACTGATTTCTACTATTTCACC*	F	RT-PCR
	OLEC10606	*GGCCTCATAAGACATTTCGG*	R	RT-PCR, SEQ
SPy_1219	OLEC10623	*GGATTGGGTACTTAATGTTGC*	F	RT-PCR, SEQ
	OLEC10624	*ATGAGCTAGTGCTTCAATAGC*	R	RT-PCR
SPy_1239 / pepN	OLEC10626	*CATGTATCGATGAACCACAAGC*	F	RT-PCR, SEQ
	OLEC10627	*CTTTCCATGAAGTTCTCCTAGC*	R	RT-PCR
SPy_1302 / amyA	OLEC10614	*CAAACCAGCAATAGTTACCAAGC*	F	RT-PCR, SEQ
	OLEC10615	*GGTAGATGAGTATCAAAGAACCAG*	R	RT-PCR
SPy_1351 / aroK	OLEC11118	*GCAAATGATAATAGTATCATCG*	F	RT-PCR, SEQ
	OLEC11119	*CCATACGTTGTTGATAAAATTC*	R	RT-PCR
SPy_1493 / fakB2	OLEC11120	*GGAGGTCGTATTGGTCGTG*	F	RT-PCR
	OLEC11121	*GAGTGGTTGTAATAAGCTGCG*	R	RT-PCR, SEQ
SPy_1584 / aroE	OLEC11130	*CTTGGCTGACGAAGGAGTGTC*	F	RT-PCR
	OLEC11131	*GGCTTCATTCCTACACTTGTAGC*	R	RT-PCR, SEQ
SPy_1846 / dinP	OLEC11624	*GGAAAGCCTGTTGTGATTGG*	F	RT-PCR
	OLEC11625	*CAGTCACATCAAGGTAAGCC*	R	RT-PCR, SEQ
SPy_1962 / proS	OLEC11126	*CTTACAAGCAATTGCCCTTAAAC*	F	RT-PCR
	OLEC11127	*CGCAATAGACTTGTCAAGAACC*	R	RT-PCR, SEQ
ermB′-A2I-*ffluc*	OLEC14185	*CATGAACAAAAATATAAAATATTCTCAAAACAGTGC*	F	RT-PCR
	OLEC14186	*GCATCTGTAAAAGCAATTGTTCC*	R	RT-PCR, SEQ
**Quantitative RT-PCR**				
SPy_0093 / adcC	OLEC14246	*ACCAAAGGCTGGACGAGTTA*	F	qRT-PCR
	OLEC14247	*CGTAAACGGTGGATGGAAAACC*	R	qRT-PCR
SPy_0098 / rpoB	OLEC13490	*CTGTCTTTGACGGGGCTTCA*	F	qRT-PCR
	OLEC13491	*ACCGGTGCGACCATCATAAA*	R	qRT-PCR
SPy_0167 / slo	OLEC13505	*ATGAAGCTCCGCCACTCTTT*	F	qRT-PCR

Purpose	Code	Sequence 5′-3′[a]	F/R[b]	Usage[c]
	OLEC13506	*TGCACTAAAGGCCGCTTCAA*	R	qRT-PCR
SPy_0209 / tadA	OLEC13939	*TCCCCATTGGCTGTGTCATT*	F	qRT-PCR
	OLEC13940	*GCCATCATTTCAGCGTGCAT*	R	qRT-PCR
SPy_0476 / era	OLEC13488	*TGACGCAACAAGAGGTTCCA*	F	qRT-PCR
	OLEC13489	*GCTATCGCGCTCCACCATAA*	R	qRT-PCR
SPy_1152 / gyrA	OLEC13482	*CCGACTGGTGCCCTTGTTAT*	F	qRT-PCR
	OLEC13483	*CGTTCCCGACCTGTTTGAGT*	R	qRT-PCR
SPy_1219	OLEC14254	*TCGTTCTCCTGGCCCTTATT*	F	qRT-PCR
	OLEC14255	*TTCCCCCTGTAGCCATAACA*	R	qRT-PCR
SPy_1239 / pepN	OLEC14256	*GCGAGGAGACTGGTCTTTGG*	F	qRT-PCR
	OLEC14257	*GAAGTTCTCCTAGCGCAAACG*	R	qRT-PCR
SPy_1302 / amyA	OLEC13507	*AGACAGTACCAGGGGAACAAC*	F	qRT-PCR
	OLEC13508	*ACTTGGCAATGGTCTGAGTGT*	R	qRT-PCR
SPy_1351 / aroK	OLEC14475	*TCTTTTGAAACTTTGTACCAGCGT*	F	qRT-PCR
	OLEC14476	*TCCCTCATAAAATACCATACGTTGT*	R	qRT-PCR
SPy_1493 / fakB2	OLEC13509	*CGCTTGTCAAAGGTCGAGGTA*	F	qRT-PCR
	OLEC13510	*CAGGCTAGCTTCACCAGCAT*	R	qRT-PCR
SPy_1584 / aroE	OLEC14473	*CGCCAAAGAGGTTCGTTTGT*	F	qRT-PCR
	OLEC14474	*GTCCTTGTTAGCTGGTTCAGTT*	R	qRT-PCR
SPy_1805 / secA	OLEC13486	*AGGGCGTCAAGTGGAATCTG*	F	qRT-PCR
	OLEC13487	*CGCTGTTCACGCATCACATC*	R	qRT-PCR
SPy_1876 / rnjA	OLEC14481	*CTTTGGCGTGAAGCAACTGT*	F	qRT-PCR
	OLEC14482	*CGACCGGTTCTGGAATGGAA*	R	qRT-PCR
SPy_1962 / proS	OLEC14264	*AGACGGCTATAGTTTCCATCACA*	F	qRT-PCR
	OLEC14265	*CGCCATCTCCAATAATCCCTTTG*	R	qRT-PCR
ffluc	OLEC14238	*AGAGATACGCCCTGGTTCCT*	F	qRT-PCR
	OLEC14239	*TGCCAACCGAACGGACATTT*	R	qRT-PCR
16S rRNA	OLEC14228	*TGAGTGCAGAAGGGGAGAGT*	F	qRT-PCR
	OLEC14229	*GAGCCTCAGCGTCAGTTACA*	R	qRT-PCR
**Probes for Northern blotting**				
SPy_t16 / tRNA$^{Ser}_{UGA}$	OLEC10579	*GCACGCTTTTACACGCCTGACG*	R	NB
SPy_t37 / tRNA$^{Arg}_{ACG}$	OLEC9362	*GAACCTCTAACCGCCTGATT*	R	NB
*SPy_*t52 / tRNA$^{Leu}_{AAG}$	OLEC9363	*CACACGACCTAAAGCGGTCAC*	R	NB
5S rRNA	OLEC288	*CTAAGCGACTACCTTATCTCA*	R	NB
**Templates for *in vitro* transcriptions**				
pEC2322	OLEC8611	AGCT**TAATACGACTCACTATAG***CGGGGATGGCGGAATTGGC*	F	OA
	OLEC8612	*AGACGCGCAGGACTAAGGATCCTGTGACCGCTTTAGGTCG*	F	OA
	OLEC8613	*TGTGGGTTCAAGTCCCACTCTCCGCACCAGG*	F	OA
	OLEC8614	*GCGTCTGCCAATTCCGCCATCCCCG***CTATAGTGAGTCGTATTA**	R	OA
	OLEC8615	*CCCACACGACCTAAAGCGGTCACAGGATCCTTAGTCCTGC*	R	OA

Purpose	Code	Sequence 5′-3′[a]	F/R[b]	Usage[c]
	OLEC8616	GATCC*CTGGTGCGGAGAGTGGGACTTGAA*	R	OA
pEC2405	OLEC8926	AGCTTAATACGACGACTCACTATA*GCACCCTTAG*	F	OA
	OLEC8927	*CTCAACTGGATAGAGTACCTGACTACGAATCAGGCGGT*	F	OA
	OLEC8928	*TAGAGGTTCGACTCCTCTAGGGTGCACCAG*	F	OA
	OLEC8929	GATC*CTGGTGCACCCTAGAGGAGTCGA*	R	OA
	OLEC8930	*ACCTCTAACCGCCTGATTCGTAGTCAGGTACTCTATCCA*	R	OA
	OLEC8931	*GTTGAGCTAAGGGTGC*TATAGTGAGTCGTCGTATTA	R	OA
	OLEC9115	**CTATAGTGAGTCGTATTA**AGCTTGGCGTAATCATGGTCATAGC	R	SDM-PCR
	OLEC9117	CCAAGCT**TAATACGACTCACTATAG***CACCCTTAGCTCAACTG*	F	SDM-PCR
pEC2406	OLEC8920	AGCTTAATACGACGACTCACTATA*GCATCCGTAGCTCAGC*	F	OA
	OLEC8921	*TGGATAGAGTACTCGGCTACGAACCGAGCGGTC*	F	OA
	OLEC8922	*GGAGGTTCGAATCCTCCCGGATGCACCAG*	F	OA
	OLEC8923	GATC*CTGGTGCATCCGGGAGGATTCGAACCTCCGACCGC*	R	OA
	OLEC8924	*TCGGTTCGTAGCCGAGTACTCTATCCAGCTGAG*	R	OA
	OLEC8925	*CTACGGATGC*TATAGTGAGTCGTCGTATTA	R	OA
	OLEC9115	**CTATAGTGAGTCGTATTA**AGCTTGGCGTAATCATGGTCATAGC	R	SDM-PCR
	OLEC9116	CCAAGCT**TAATACGACTCACTATAG***CATCCGTAGCTCAGC*	F	SDM-PCR
**TadA protein production**				
pEC2360	OLEC8780	AGCACAT*ATGCCATATAGTTTAGAAGAGC*	F	PCR
	OLEC8782	ACGTAAGCTT*GTCAAAGGGATCTGACTG*	R	PCR
pEC2389	OLEC8956	GATCCAT*ATGTCTGAAGTCGAATTTAGCCACG*	F	PCR
	OLEC8958	AGCGAAGCTT*ATCCGTCGAGGATTGCGC*	R	PCR
**Ectopic expression of *tadA* in *S. pyogenes***				
pEC2173 linearisation	OLEC11689	AAGGCCCACTTTTGTGGGCCTTTTTT*GGATCGGCCGCTCTAGAGTC*	F	PCR
	OLEC11694	*GAGCTCCAATTCGCCCTATAGTGAG*	R	PCR
pEC2812	OLEC11695	CTATAGGGCGAATTGGAGCTC*GCCTATCATTTTCAATGAAAGAAGTC*	F	PCR
	OLEC11696	AAGGCCCACAAAAGTGGGCCTTTTTT*TAAAAAATGCCCCTTTTCTTCAC*	R	PCR
pEC2813	OLEC11695	CTATAGGGCGAATTGGAGCTC*GCCTATCATTTTCAATGAAAGAAGTC*	F	PCR
	OLEC11697	CTAAACTATATGGCATT*AAAAAATGCCCCTTTTCTTCACTG*	R	PCR
	OLEC11692	AAGGCCCACAAAAGTGGGCCTTTTTT*CTAATGGTGATGATGGTGATGAGAACCTCC*	R	PCR
	OLEC11698	GGGCATTTTTTA*ATGCCATATAGTTTAGAAGAGCAAACTTATTTC*	F	PCR
	OLEC11541	*GTCAAAGGGATCTGACTGTTC*	R	PCR

Purpose	Code	Sequence 5′-3′[a]	F/R[b]	Usage[c]
	OLEC11059	CAAGGAACAGTCAGATCCCTTTGACGGTGGAGGTTCTCATCACCATCATCACCATTAGAAAAAAGGCCCACTTTTGTGGGCCTTTTTTTAACGCTGATAGTGCTAGTGTAG	F	OA
	OLEC11060	CTACACTAGCACTATCAGCGTTAAAAAAAGGCCCACAAAAGTGGGCCTTTTTTCTAATGGTGATGATGGTGATGAGAACCTCCACCGTCAAAGGGATCTGACTGTTCCTTG	R	OA
***ermBL*-based editing reporter assay**				
ermB(5′UTR)-*ermB′*:A2I (for pEC3045)	OLEC14013	CCTAAATTATGGTACAATGTAAGAGGAAGTTAAATTAGATGCTAAAAATTTGTAATTAAGAAGGAGGGATTCGTCATGTTGGTATTCCAAATGCGTAATGTAGATAAAACAT	F	OA
	OLEC14014	AGTCATAAGATTAGTCACTGGTAGGAATTAATCTAACGTATTTATTTATCTGCGTAATCACTGTTTTTAGTCTGTTTCAAAACAGTAGATGTTTTATCTACATTACGCATTTGGAATA	R	OA
	OLEC14015	AATTCCTACCAGTGACTAATCTTATGACTTTTTAAACAGATAACTAAAATTACAAACAAATCGTTTAACTTCTGTATTTATTTATAGATGTAATCACTTCAGGAGTGATTACATGAAC	F	OA
	OLEC14016	CTTTCTTTATGTTTTTGGCGTCTTCAGAACCTCCACCAGTACGCTTCGTAGGCGCACTGTTTTGAGAATATTTTATATTTTTGTTCATGTAATCACTCCTGAAGTGATTACA	R	OA
	OLEC14017	*CCTAAATTATGGTACAATGTAAGAGGAAGT*	F	PCR
	OLEC14018	*CTTTCTTTATGTTTTTGGCGTCTTCAG*	R	PCR
ffluc (for pEC3045)	OLEC14009	*GAAGACGCCAAAAACATAAAGAAAGG*	F	PCR
	OLEC14010	*TTACAATTTGGACTTTCCGCCC*	R	PCR
TT3 (for pEC3045)	OLEC14011	GAAGGGCGGAAAGTCCAAATTGTAATAGAAAAAAGGCCCACTTTTGTGGGCCTTTTTTGGATCGGCCGCTCTAGAGTCGTGTT	F	OA
	OLEC14012	AACACGACTCTAGAGCGGCCGATCCAAAAAAGGCCCACAAAAGTGGGCCTTTTTTCTATTACAATTTGGACTTTCCGCCCTTC	R	OA
ermB(5′UTR)-*ermB′*:A2I:*ffluc*-TT3 (for pEC3045)	OLEC14017	*CCTAAATTATGGTACAATGTAAGAGGAAGT*	F	PCR
	OLEC13810	*CGACTCTAGAGCGGCCGATC*	R	PCR
pEC2173 backbone with P_{gyrA}	OLEC10676	*GGATCGGCCGCTCTAGAG*	F	PCR
	OLEC14008	*CTCTTACATTGTACCATAATTTAGGTAAAATTGC*	R	PCR
pEC3046	OLEC14019	*GGATTCGTC*TAATTAG*TATTCCAAATGCGTAATGTAGATAAAACATCTACTG*	F	SDM-PCR
	OLEC14020	*TTGGAATAC*TAATTAG*ACGAATCCCTCCTTCTTAATTACAAATTTTTAGC*	R	SDM-PCR
pEC3047	OLEC14135	*CAAAAATATAAAATATTCTCAAAACAGTGC*	F	PCR
	OLEC14136	*CTGAAGTGATTACATCTATAAATAAATACAG*	R	PCR

Purpose	Code	Sequence 5′-3′[a]	F/R[b]	Usage[c]
	OLEC14163	*AGGGATTCGTCATGAACAAAAATATAAAATATTCTC AAAACAGTGCG*	F	SDM-PCR
	OLEC14164	*TATTTTTGTTCATGACGAATCCCTCCTTCTTAATTAC AAATTTTTAG*	R	SDM-PCR
pEC3069	OLEC14216	*AGGGATTCGTCTAGAACAAAAATATAAAATATTCTC AAAACAGTGCG*	F	SDM-PCR
	OLEC14217	*TATTTTTGTTCTAGACGAATCCCTCCTTCTTAATTAC AAATTTTTAG*	R	SDM-PCR
**Suicide vector template for deletion of *tadA* from genome (pEC2899)**				
pEC801 linearisation	OLEC9943	*CTGCAGGCATGCAAGCTTGCG*	F	PCR
	OLEC9944	*TCTAGAGGATCCCCGGGTACCGAG*	R	PCR
Upstream fragment	OLEC12752	ACCCGGGGATCCTCTAGAC*TGTGGCAGCCTAAAGT ATTGC*	F	PCR
	OLEC12753	TGTATGCTATACGAACGGTA*TCAAGAAAGACTCCCC AAGTTCC*	R	PCR
Downstream fragment	OLEC12754	GCATACATTATACGAACGGTA*AGTTTTAAGGAGTTT GTTATCACATGC*	F	PCR
	OLEC12755	AAGCTTGCATGCCTGCAG*TCAGATTGAGCACATCTA ATGCTTG*	R	PCR
lox71-$P_{ermAM/B}$-*ermAM/B*-lox66	OLEC1943	*TACCGTTCGTATAGCATACATTATACGAAGTTATCC GTAGCGGTTTTCAAAATTTGCAACC*	F	PCR
	OLEC1932	*TACCGTTCGTATAATGTATGCTATACGAAGTTATTTA TTTCCTCCCGTTAAATAATAGATAACTATTAAA*	R	PCR
PCR ligation	OLEC11417	*ACCCGGGGATCCTCTAGA*	F	LM-PCR
	OLEC11418	*AAGCTTGCATGCCTGCAG*	R	LM-PCR
Validation of locus	OLEC12750	*GGATGCAGTGATTATTAGCC*	F	SEQ
	OLEC12751	*GATATACTAACACAATTGAAGGC*	R	SEQ
**Suicide vector template for genomic integration of AHT-inducible promoter (pEC2901)**				
pEC801 linearisation	OLEC9943	*CTGCAGGCATGCAAGCTTGCG*	F	PCR
	OLEC9944	*TCTAGAGGATCCCCGGGTACCGAG*	R	PCR
cat86-P_{tet}	OLEC12291	*GTCAATATTTTTTTTAGTTTTTCATGAACTCG*	R	PCR
	OLEC12294	*CGAAAATTGGATAAAGTGGGATATTTTT*	F	PCR
TT3 insertion upstream of *cat86*	OLEC12295	GGTACCCGGGGATCCTCTAGATGTACAAAAAAGGC CCACAAAAGTGGGCCTTTTTTCGAAAATTGGATAAA GTGGGATAT	F	OA
	OLEC12296	ATATCCCACTTTATCCAATTTTCGAAAAAAGGCCCA CTTTTGTGGGCCTTTTTTGTACATCTAGAGGATCCCC GGGTACC	R	OA
P_{tet}	OLEC12292	CTCGAGTTCATGAAAAACTAAAAAAAATATTGACAC TCTATCATTGATAGAGTATAATTAAAATAAGACTCTA TCATTGATAGAGTGTACAGTCGACCTGCAGGCATGC AAGCTTGCG	F	OA
	OLEC12293	CGCAAGCTTGCATGCCTGCAGGTCGACTGTACACTC TATCAATGATAGAGTCTTATTTTAATTATACTCTATC AATGATAGAGTGTCAATATTTTTTTTAGTTTTTCATGA ACTCGAG	R	OA
Sequencing of *cat86*-P_{tet} cassette	OLEC12291	*GTCAATATTTTTTTTAGTTTTTCATGAACTCG*	R	SEQ
	OLEC12294	*CGAAAATTGGATAAAGTGGGATATTTTT*	F	SEQ

Purpose	Code	Sequence 5′-3′[a]	F/R[b]	Usage[c]
**EC2353 (SF370 P_{tet}-*rnjA*) via pEC812 and pEC852**				
tetR(A582C) (pEC812)	OLEC3322	*CCGCAGATGATCAATTCAAGG*	F	SDM-PCR
	OLEC3323	*CCTTGAATTGATCATCTGCGG*	R	SDM-PCR
Upstream fragment (pEC852)	OLEC3524	AAAAACTGCAG*TAGTGAAGCATGGGCTTGTG*	F	PCR
	OLEC3525	AAAAACTGCAG*CTTAGAACTCCGTTAATTCAAAAACACC*	R	PCR
Downstream fragment (pEC852)	OLEC3526	AAAACATATG*ACAAATATCAGTTTAAAACCTAATGAAGTTG*	F	PCR
	OLEC3527	AAAAACATATG*CTGCCATAGACTCACCTTGACTACC*	R	PCR
**EC3453 (SF370 P_{tet}-*slo*) via pEC2964**				
Upstream fragment (pEC2964)	OLEC12908	TACCCGGGGATCCTCTAGAT*CCGTGCTATCTTGTTGTCTATGG*	F	PCR
	OLEC12909	CACTTTTGTGGGCCTTTTTT*AAGTAAACTATACATTAGCAAATAGTTTTTGTC*	R	PCR
Downstream fragment (pEC2964)	OLEC12910	AATAAGACTCTATCATTGATAGAGT*AAAAATAATATAAGGTGGTTTACATGAGAAAC*	F	PCR
	OLEC12911	TGCATGCCTGCAGGTCGACT*GATGGACCTCTGTTACCTCAATATC*	R	PCR
Validation of locus	OLEC12912	*GGTTGAAATGGTCATGACAGATG*	F	PCR, SEQ
	OLEC12913	*TCAGCATACTTGTCGGCAG*	R	PCR, SEQ
**EC3456 (SF370 P_{tet}-*amyA*) via pEC2965**				
Upstream fragment (pEC2965)	OLEC12896	TACCCGGGGATCCTCTAGAT*GACAACGAATTGCTAATCAGCTG*	F	PCR
	OLEC12897	CACTTTTGTGGGCCTTTTTT*AAGTTAAGTTTATCGTAATCGCTTTCATTTG*	R	PCR
Downstream fragment (pEC2965)	OLEC12898	AATAAGACTCTATCATTGATAGAGT*AAACGTTTAAGTTTCGAAACTATTACTG*	F	PCR
	OLEC12899	TGCATGCCTGCAGGTCGACT*CAAAGTTTCAATTACGTCTGGTG*	R	PCR
Validation of locus	OLEC12900	*CTTACCTAGATGCTCGTCG*	F	PCR, SEQ
	OLEC12901	*CCGGCAATTAAACCATAACC*	R	PCR, SEQ
**EC3459 (SF370 P_{tet}-*fakB2*) via pEC2966**				
Upstream fragment (pEC2966)	OLEC12891	TACCCGGGGATCCTCTAGAT*GAGGACATTTAGATAACAAACTGGACG*	F	PCR
	OLEC12892	CACTTTTGTGGGCCTTTTTT*CTCTTTATTCTAACACTCCTTACTAAATAAAG*c	R	PCR
Downstream fragment (pEC2966)	OLEC12893	AATAAGACTCTATCATTGATAGAGT*GTGAGAAACTAGTTTAGAATGGAAAAATAC*	F	PCR
	OLEC12894	TGCATGCCTGCAGGTCGACT*CATTAAGTAGCGAGCTCAAAACAC*	R	PCR
Validation of locus	OLEC12895	*GAAGATACAATTGAGCCAGAGC*	F	PCR, SEQ
	OLEC11121	*GAGTGGTTGTAATAAGCTGCG*	R	PCR, SEQ
**EC3570 (SF370 P_{tet}-TT_{tadA}) via pEC3000**				
cat86-P_{tet}	OLEC12294	*CGAAAATTGGATAAAGTGGGATATTTTT*	F	PCR, SEQ
	OLEC12869	*ATGAGATCACCTCCTTAACTAGAC*	R	PCR, SEQ
Upstream fragment	OLEC12872	ACCCGGGGATCCTCTAGAC*GTGAAGAGAGTAACCAAGCC*	F	PCR

Purpose	Code	Sequence 5′-3′[a]	F/R[b]	Usage[c]
(pEC2913)	OLEC12868	AATATCCCACTTTATCCAATTTTCG*GTTTTCATCTCGATTGGGTCTGA*	R	PCR
Downstream fragment (pEC2913)	OLEC12873	CTAGTTAAGGAGGTGATCTCAT*AGTTTTAAGGAGTTTGTTATCACATGC*	F	PCR
	OLEC12755	AAGCTTGCATGCCTGCAG*TCAGATTGAGCACATCTAATGCTTG*	R	PCR
PCR ligation (up, cassette, down)	OLEC11417	ACCCGGGGATCCTCTAGA	F	LM-PCR
	OLEC11418	AAGCTTGCATGCCTGCAG	R	LM-PCR
RBS mutagenesis (pEC3000)	OLEC13498	TTTCCTC*TAACTAGACTCGAAGATCTATTCGAG*	R	SDM-PCR
	OLEC13499	*TCGAGTCTAGTTA*GAGGAAA*AGTTTTAAGGAGTTTGTTATCACATGC*	F	SDM-PCR
**EC3576 (SF370 P_{tet}-*tadA*^{low}) via pEC3001**				
cat86-P_{tet}	OLEC12294	*CGAAAATTGGATAAAGTGGGATATTTTT*	F	PCR, SEQ
	OLEC12869	*ATGAGATCACCTCCTTAACTAGAC*	R	PCR, SEQ
Upstream fragment (pEC2914)	OLEC12752	ACCCGGGGATCCTCTAGA*CTGTGGCAGCCTAAAGTATTGC*	F	PCR
	OLEC12866	CAAACTCCTTAAAACT*CAAGAAAGACTCCCCAAGTTCC*	R	PCR
TT_{tadA}	OLEC12867	GAGTCTTTCTTGA*GTTTTAAGGAGTTTGTTATCACATGC*	F	PCR
	OLEC12868	AATATCCCACTTTATCCAATTTTCG*GTTTTCATCTCGATTGGGTCTGA*	R	PCR
Downstream fragment (pEC2914)	OLEC12870	CTAGTTAAGGAGGTGATCTCAT*ATGCCATATAGTTTAGAAGAGCAAAC*	F	PCR
	OLEC12871	AAGCTTGCATGCCTGCAG*CTAGTCAAAGGGATCTGACTGTTC*	R	PCR
PCR ligation (up, cassette, down)	OLEC11417	ACCCGGGGATCCTCTAGA	F	LM-PCR
	OLEC11418	AAGCTTGCATGCCTGCAG	R	LM-PCR
RBS mutagenesis (pEC3001)	OLEC13498	TTTCCTC*TAACTAGACTCGAAGATCTATTCGAG*	R	SDM-PCR
	OLEC13500	*TCGAGTCTAGTTA*GAGGAAA*ATGCCATATAGTTTAGAAGAGCAAAC*	F	SDM-PCR
**EC3622 (SF370 P_{tet}-*tadA*) via pEC3021**				
RBS mutagenesis (pEC3021)	OLEC13766	T*TTTTCCTCTAACTAGACTCGAAGATCTATTCGAGC*	R	SDM-PCR
	OLEC13767	*TCGAGTCTAGTTAGAGGAAAAAATGCCATATAGTTTAGAAGAGCAAAC*	F	SDM-PCR
**Verification of virulence regulator gene integrity in mutant strains**				
covRS	OLEC4856	*TCGCTAGAAGACTATTTGACCAT*	F	PCR, SEQ
	OLEC4867	*AAGACATCGCGATTGACAGT*	R	PCR, SEQ
	OLEC3609	*GGCTATGTTCAAGTCTTTCATG*	F	SEQ
	OLEC3610	*CCAAATAACTCAACAACTAGTAGC*	R	SEQ
mga	oliRN172	*AGTTGACTAACCAATTGATCTACGCCTTTT*	F	PCR, SEQ
	OLEC290	*TTAACCTCTGTTTGATTCGC*	R	PCR, SEQ
ropB	OLEC4854	*AGCGACTATCATCCGAAACAT*	F	PCR, SEQ
	OLEC4855	*GCCCTGGAGCTGTTGAGATA*	R	PCR, SEQ

[a] *italic*, sequence annealing to the template; underlined, restriction site; **bold**, T7 promoter.

[b] F, forward primer; R, reverse primer.

[c] LM-PCR, PCR-mediated ligation; NB, probe for Northern blot; SEQ, sequencing; SDM-PCR, PCR-mediated site-directed mutagenesis; RT-PCR, reverse transcription-PCR; OA, oligo assembly

Table S3. Plasmids used in this study.

Plasmids	Relevant characteristics	Source
**Vector backbones for *S. pyogenes***		
pLZ12Km2-P*23R*:TA:*ffluc* (pEC2173)	pSH71, *aphIII*, ω-ε-ζ TA cassette, P_{23R}-*ffluc*	Addgene #88900 (20)
**Deletion of *tadA***		
pEC801	pRO1600/ColE1, *bla*	SEVA (21)
pEC2899	pEC801Ω*tadA*(up)-lox71-$P_{ermAM/B}$-*ermAM/B*-lox66-*tadA*(down)	This study
**Integration of AHT-inducible promoter into the genome of *S. pyogenes***		
pEC536	ColE1-*repDEG*, *cat86*, P_{tet}	pEU8517 (2)
pEC808	pEC801Ω*cat86*-P_{tet}	This study
pEC812	pEC801Ω*cat86*-P_{tet}*	This study
pEC852	pEC801Ω*rnjA*(up)-*cat86*-P_{tet}*-*rnjA*(1..925)	This study
pEC2901	pEC801ΩTT3-*cat86*-P_{tet}	This study
pEC2964	pEC801ΩP_{nga}(up)-TT3-*cat86*-P_{tet}-P_{nga}(down)	This study
pEC2965	pEC801ΩP_{malX}(up)-TT3-*cat86*-P_{tet}-P_{malX}(down)	This study
pEC2966	pEC801ΩP_{fakB2}(up)-TT3-*cat86*-P_{tet}-P_{fakB2}(down)	This study
pEC2913	pEC801Ω*tadA*-TT3-*cat86*-P_{tet}-*tadA*(down)	This study
pEC2914	pEC801Ω*tadA*(up)-TT3-*cat86*-P_{tet}-*tadA*	This study
pEC3000	pEC801Ω*tadA*-TT3-*cat86*-P_{tet}(RBSmut)-*tadA*(down)	This study
pEC3001	pEC801Ω*tadA*(up)-TT3-*cat86*-P_{tet}(RBSmut)-*tadA*	This study
pEC3021	pEC801Ω*tadA*(up)-TT3-*cat86*-P_{tet}(RBSmut2)-*tadA*	This study
**TadA protein production**		
pET-21a(+)	pBR322, *bla*, *lacI*, P_{T7}(lacO)	Novagen
pEC2360	pET-21a(+)Ω*tadA*(*Spy*)	This study
pEC2389	pET-21a(+)Ω*tadA*(*Eco*)	This study
***In vitro* transcription**		
pUC19	pMB1, *bla*, P_{lac}(lacO)-*lacZα*	NEB
pEC2322	pUC19ΩP_{T7}-tRNA-Leu-AAG(*Spy*)	This study
pEC2405	pUC19ΩP_{T7}-tRNA-Arg-ACG(*Spy*)	This study
pEC2406	pUC19ΩP_{T7}-tRNA-Arg-ACG(*Eco*)	This study
**Ectopic expression of *tadA***		
pEC2812	pLZ12Km2-TA:P_{gyrA}(*Sag*)-TT3	This study
pEC2813	pLZ12Km2-TA:P_{gyrA}(*Sag*)-*tadA*(*Spy*):His_6-TT3	This study

**_ermBL_ reporter assay**		
pEC3045	pLZ12Km2-TA:P_{gyrA}(*Sag*)-*ermBL*-*ermB*':A2I:*ffluc*-TT3	This study
pEC3046	pLZ12Km2-TA:P_{gyrA}(*Sag*)-*ermBL*(M1*)-*ermB*':A2I:*ffluc*-TT3	This study
pEC3047	pLZ12Km2-TA:P_{gyrA}(*Sag*)-*ermB*':A2I:*ffluc*-TT3	This study
pEC3069	pLZ12Km2-TA:P_{gyrA}(*Sag*)-*ermB*'(M1*):A2I:*ffluc*-TT3	This study

Promoters are denoted as P with the related gene name. The erythromycin resistance cassette ($P_{ermAM/B}$-*ermAM/B*) is flanked by lox71 and lox66 sites for Cre recombinase-based excision. The AHT-inducible P_{tet} cassette harbours the repressor *tetR* and two divergent promoters with three operator sites, and is separated from the chloramphenicol resistance gene *cat86* by two T4 terminators as previously described (1, 2). A mutant version of P_{tet} with *tetR*(A582C) is shown as P_{tet}*. Mutations of the ribosomal binding site as shown in Figure S1 are described as P_{tet}(RBSmut) and P_{tet}(RBSmut2). Transcriptional terminators are denoted as TT, with TT3 as the phage T3 terminator. Species are indicated in brackets after the respective genes if applicable (*Eco*: *E. coli*; *Sag*: *S. agalactiae*; *Spy*: *S. pyogenes*).

Table S4. Efficiency of qRT-PCR primers.

Gene name	Locus tag	Primer set	Amplicon length	PCR efficiency	% efficiency
**Reference genes**					
rpoB	*SPy_0098*	OLEC13490+13491	101 bp	1.984	98.4
era	*SPy_0476*	OLEC13488+13489	113 bp	1.948	94.8
gyrA	*SPy_1152*	OLEC13482+13483	119 bp	2.032	103.2
secA	*SPy_1805*	OLEC13486+13487	99 bp	1.983	98.3
16S rRNA	-/-	OLEC14228+14229	112 bp	2.012	101.2
**Target genes**					
adcC	*SPy_0093*	OLEC14246+14247	119 bp	2.002	100.2
slo	*SPy_0167*	OLEC13505+13506	107 bp	1.962	96.2
tadA	*SPy_0209*	OLEC13939+13940	103 bp	1.991	99.1
-/-	*SPy_1219*	OLEC14254+14255	92 bp	1.974	97.4
pepN	*SPy_1239*	OLEC14256+14257	81 bp	1.902	90.2
amyA	*SPy_1302*	OLEC13507+13508	114 bp	2.025	102.5
aroK	*SPy_1351*	OLEC14475+14476	123 bp	1.925	92.5
fakB2	*SPy_1493*	OLEC13509+13510	119 bp	1.983	98.3
aroE	*SPy_1584*	OLEC14473+14474	90 bp	2.041	104.1
rnjA	*SPy_1876*	OLEC14481+14482	109 bp	2.044	104.4
proS	*SPy_1962*	OLEC14264+14265	125 bp	2.043	104.3
ffluc	-/-	OLEC14238+14239	101 bp	1.947	94.7

Table S5. Differential expression of selected oxidative stress signature genes upon exposure to H_2O_2.

			0.5 mM H_2O_2		1.0 mM H_2O_2	
**Locus tag**	**Gene**	**Function**	**15 min**	**30 min**	**15 min**	**30 min**
**Peroxide detoxification**						
SPy_1406	*sodA*	superoxide dismutase	2.39	1.92	2.86	2.72
SPy_2079	*ahpC*	alkyl hydroperoxide reductase C	1.48	1.27	2.07	1.58
SPy_2080	*ahpF*	alkyl hydroperoxide reductase F	1.33	1.41	1.80	1.57
**DNA repair and protein quality control**						
SPy_0185	*polA*	DNA damage-induced DNA polymerase I	1.18	n.s.	1.93	1.42
SPy_2216	*htrA*	membrane-anchored quality control protease	n.s.	n.s.	1.03	1.39
**Metal ion homeostasis**						
SPy_0453	*mtsA*	metal ABC transporter subunit	n.s.	n.s.	–1.15	–1.28
SPy_1434	*zntA*	heavy metal transporter ATPase	–1.38	–2.10	n.s.	–1.72
SPy_1531	*dpr*	ferritin-like iron-binding protein	2.58	1.59	3.17	3.09
SPy_1798	*shr*	heme-binding protein	n.s.	n.s.	–1.26	–1.60

Gene expression for each time point and H_2O_2 concentration was compared relative to the mock-treated control. Genes were considered differentially expressed with an absolute log_2 fold change of at least 1 and an adjusted p value below 0.05. n.s.: not significant. Compare Table S12 for full differential expression analysis.

Table S6. Possible effects of editing-mediated recoding in *S. pyogenes*.

Locus tag (gene)	UniProt ID	Gene function	Re-coding	PROVEAN score	Protein structure(s)	Location of recoded residue	Function of residue or protein domain	Possible effect of recoding
SPy_0442 (*glpT*)	Q9A164	glycerol-3-phosphate permease	T14A	0.424 (neutral)	1PW4 (*E. coli*)	transmembrane helix 1	TM helices 1 and 7 with major conformational changes during substrate translocation (22)	effect on helix movement and flexibility?
SPy_0458 (*ftsK*)	Q9A155	DNA segregation ATPase	K13E	-2.233 (neutral)	no, only motor domain	N-terminal integral membrane domain	domain required for septum recruitment via interaction with early division proteins (23, 24)	altered septum recruitment dynamics?
SPy_0477	Q9A141	putative Nudix hydrolase	Y103C	-4.960 (deleterious)	no	Nudix hydrolase domain	residue not part of conserved NUDIX box motif, substrate unknown	unclear
SPy_0711 (*speC*)	P0C0I5	streptococcal pyro-genic exotoxin C	K30E	0.417 (neutral)	1AN8 and 1HQR (*S. pyogenes*)	close to signal peptide cleavage site	residue not involved in immunogenicity (25) or dimerization (26)	interference with SpeC export?
SPy_1094 (*pplD*)	Q99ZT1	cell wall deacetylase	K208E	0.886 (neutral)	6DQ3 (*S. pyogenes*)	NodB homology domain	no direct involvement in catalytic activity (27)	unclear
SPy_1134 (*egtUBC*)	Q99ZQ2	ergothioneine permease/substrateb inding protein	K317E	0.915 (neutral)	7TXK (*S. pneu-moniae*), 6EYQ (*B. subtilis*)	type 2 periplasmic binding fold (substrate-binding domain)	residue not directly involved in substrate binding (28, 29)	effect on structural change upon substrate binding?
SPy_1173 (*gid*)	Q99ZL9	tRNA-U54 methyltransferase	K6E	-0.024 (neutral)	3G5R (*T. thermo-philus*)	N terminal extension of *S. pyogenes* TrmFO	role of extension at N terminus unknown	unclear

Locus tag (gene)	UniProt ID	Gene function	Re-coding	PROVEAN score	Protein structure(s)	Location of recoded residue	Function of residue or protein domain	Possible effect of recoding
SPy_1302 (*amyA*)	Q99ZB3	cyclodextrin glycosyltransferase	K644E	0.577 (neutral)	1CXH (*N. circulans*), 1AC0 (*A. niger*)	carbohydrate-binding module 20 (CBM20)	CBM20 required for starch binding, but residue not part of consensus binding sites (30, 31)	altered substrate specificity or affinity?
SPy_1351 (*aroK*)	Q99Z84	shikimate kinase	K124E	-2.155 (neutral)	2IYZ (*M. tubercu-losis*)	C-terminal to flexible, active site-covering lid domain	residue not directly involved in nucleotide or shikimate binding (32)	Effect on lid domain movement?
SPy_1493 (*fakB2*)	P67372	fatty acid kinase binding protein	T210A	-1.118 (neutral)	6DJ6 (*S. pneu-moniae*), 7W7H (*S. suis*)	DegV domain	residue not involved in fatty acid binding or FakA interaction (33, 34)	unclear
SPy_1543 (*arcD*)	Q99YT7	putative arginine/ ornithine antiporter	K41E	-0.420 (neutral)	no	extracellular loop region	no details on substrate binding and trans-location available so far	altered binding and transport dynamics of arginine?
SPy_1708 (*lacA.1*)	Q99YH1	galactose-6-phosphate isomerase	Y64C	-8.322 (deleterious)	4LFL (*L. rhamnosus*)	occlusion of second possible binding pocket in LacAB hetero-dimer (35)	*lac.1* operon with regulatory function in *S. pyogenes* (36), but residue probably not involved in Gal6P binding even if metabolically active (35)	altered regulatory function?
SPy_1846 (*dinP*)	Q99Y66	DNA polymerase IV	K73E	-1.003 (neutral)	4R8U (*E. coli*), 4IRK (*E. coli*)	UmuC domain	residue located close to template DNA strand but without direct DNA interaction in *E. coli* (37)	altered DNA binding and translesion synthesis?

Locus tag (gene)	UniProt ID	Gene function	Re-coding	PROVEAN score	Protein structure(s)	Location of recoded residue	Function of residue or protein domain	Possible effect of recoding
SPy_1900 (thiD)	Q99Y29	hydroxy-/ phosphomethyl-pyrimidine kinase	T67A	0.330 (neutral)	1JXI (*S. typhi-murium*), 1UB0 (*T. thermophilus*)	close to dimer interface	residue not involved in substrate or ATP binding, but close to homodimer interface (38, 39)	altered oligomerization dynamics?
SPy_1941 (*cysS*)	Q99XZ9	cysteinyl-tRNA synthetase	Y135C	-8.998 (deleterious)	1L17 (*E. coli*), 1U0B (*E. coli*)	part of connective polypeptide (CP) domain inserted into Rossmann fold domain	CP domain required for tRNA acceptor end recognition, but residue not directly involved in tRNA binding (40, 41)	effect on aminoacylation efficiency?
SPy_1962 (*proS*)	Q99XY4	prolyl-tRNA synthetase	Y181C	-8.804 (deleterious)	2J3M (*E. faecalis*), 5ZNJ (*S. aureus*)	part of conserved proline binding loop anchoring motif	residue not involved in ATP or proline binding, but in anchoring of proline binding loop (42)	altered proline affinity and specificity?
SPy_2055 (*pflA*)	Q99XS7	putative pyruvate formate-lyase activating enzyme	Y81C	-8.847 (deleterious)	3CD8 (*E. coli*), 8FSI (*E. coli*)	stabilization of beta sheets of AdoMet radical core fold	residue not directly involved in SAM, FeS cluster or peptide substrate binding (43)	altered peptide substrate affinity by core fold re-modelling?
SPy_2118 (*tag*)	Q99XP0	DNA-3-methyladenine glycosylase I	K9E	-0.232 (neutral)	4AIA (*S. aureus*), 2OFI (*S. typhi*)	close to 3-methyl-adenine (3mA)-interacting Trp6	residue not involved in DNA or 3mA binding (44, 45)	Indirect influence on Trp6-3mA interaction?

For each recoding event in *S. pyogenes* SF370, locus tag, gene name, UniProt ID and gene function are listed. The putative effect of protein recoding was predicted using PROVEAN (46), and scores below -2.5 are typically considered deleterious for protein function. Selected publicly available protein structures of similar or related protein (domains) are listed with the corresponding species. Further, the location of the recoded residue within the protein, the function of the residue or the residue-containing protein domain, and the hypothetical effects of recoding are described.

SUPPLEMENTARY TABLE LEGENDS

Table S7. A-to-I editing in the transcriptome of *S. pyogenes SF370*. Identified A-to-I editing sites were grouped according to their potential effect on the coding sequence (synonymous *vs*. non-synonymous). For each genomic position, locus tag, name (if available), function of the affected gene and the encoded amino acid are shown with editing levels (in %) for each replicate and growth phase. '—' indicates that editing levels could not be determined.

Table S8. A-to-I editing in the transcriptome of *S. pyogenes* SF370 upon ectopic *tadA* overexpression. Experiments were performed as described for Figure S5 and data are presented as for Table S7.

Table S9. A-to-I editing in the transcriptomes of *S. pyogenes* 5448 and 5448AP. A-to-I editing sites in the transcriptomes of *S. pyogenes* 5448 and 5448AP were identified in duplicates for three growth phases. Data are presented as in Table S7 with the corresponding locus tag in strains 5448 and SF370 for better comparison.

Table S10. A-to-I editing in the transcriptome of *S. pyogenes* SF370 in three different culture media. *S. pyogenes* SF370 was grown to mid-logarithmic growth phase in chemically defined medium (CDM), THY and C medium in triplicate, and data are presented as in Table S7.

Table S11. A-to-I editing in the transcriptome of *S. pyogenes* SF370 in response to hydrogen peroxide. *S. pyogenes* SF370 was grown to mid-logarithmic growth phase in C medium and exposed to 0.5 mM and 1.0 mM H_2O_2 with water as mock control (0.0 mM) for 15 min and 30 min. Data are presented as in Table S7.

Table S12. Genes differentially expressed in the transcriptome of *S. pyogenes* SF370 in response to hydrogen peroxide. Experiment was performed as described for Table S11, and differential expression analysis was performed with mock-treated samples as controls for each time point and H_2O_2 concentration. Log_2 fold changes and adjusted p-values are indicated for all significant hits (adjusted p-value < 0.05 and an absolute log_2 fold change of at least 1). n.s.: not significant.

Table S13. A-to-I editing in the transcriptome of *S. pyogenes SF370* P_{tet}-*rnjA* in the presence and absence of AHT. *S. pyogenes SF370* P_{tet}-*rnjA* was grown in THY supplemented with ("induced") or without ("uninduced") 0.1 ng/mL AHT to mid-logarithmic growth phase. Data are presented as in Table S7.

SUPPLEMENTARY REFERENCES

1. Geissendörfer,M. and Hillen,W. (1990) Regulated expression of heterologous genes in *Bacillus subtilis* using the Tn*10* encoded *tet* regulatory elements. Appl. Microbiol. Biotechnol., 33, 657–663.
2. Bugrysheva,J.V. and Scott,J.R. (2010) The ribonucleases J1 and J2 are essential for growth and have independent roles in mRNA decay in *Streptococcus pyogenes*: RNases J1/J2 in *S. pyogenes*. Mol. Microbiol., 75, 731–743.
3. Le Rhun,A., Lécrivain,A.-L., Reimegård,J., Proux-Wéra,E., Broglia,L., Della Beffa,C. and Charpentier,E. (2017) Identification of endoribonuclease specific cleavage positions reveals novel targets of RNase III in *Streptococcus pyogenes*. Nucleic Acids Res., 45, 2329–2340.
4. Sarkar,P. and Sumby,P. (2017) Regulatory gene mutation: a driving force behind group a *Streptococcus* strain- and serotype-specific variation. Mol. Microbiol., 103, 576–589.
5. Sampson,J.R. and Uhlenbeck,O.C. (1988) Biochemical and physical characterization of an unmodified yeast phenylalanine transfer RNA transcribed *in vitro*. Proc. Natl. Acad. Sci. U.S.A., 85, 1033–1037.
6. Pfaffl,M.W. (2001) A new mathematical model for relative quantification in real-time RT-PCR. Nucleic Acids Res., 29, e45.
7. Vandesompele,J., De Preter,K., Pattyn,F., Poppe,B., Van Roy,N., De Paepe,A. and Speleman,F. (2002) Accurate normalization of real-time quantitative RT-PCR data by geometric averaging of multiple internal control genes. Genome Biol., 3, research0034.1.
8. Ye,J., Coulouris,G., Zaretskaya,I., Cutcutache,I., Rozen,S. and Madden,T.L. (2012) Primer-BLAST: A tool to design target-specific primers for polymerase chain reaction. BMC Bioinformatics, 13, 134.
9. Rocha,D.J.P., Santos,C.S. and Pacheco,L.G.C. (2015) Bacterial reference genes for gene expression studies by RT-qPCR: survey and analysis. Antonie van Leeuwenhoek, 108, 685–693.
10. Bar-Yaacov,D., Mordret,E., Towers,R., Biniashvili,T., Soyris,C., Schwartz,S., Dahan,O. and Pilpel,Y. (2017) RNA editing in bacteria recodes multiple proteins and regulates an evolutionarily conserved toxin-antitoxin system. Genome Res., 27, 1696–1703.

11. Martin,M. (2011) Cutadapt removes adapter sequences from high-throughput sequencing reads. EMBnet.journal, 17, 10.
12. Li,H. and Durbin,R. (2009) Fast and accurate short read alignment with Burrows-Wheeler transform. Bioinformatics, 25, 1754–1760.
13. Li,H., Handsaker,B., Wysoker,A., Fennell,T., Ruan,J., Homer,N., Marth,G., Abecasis,G., Durbin,R., and 1000 Genome Project Data Processing Subgroup (2009) The Sequence Alignment/Map format and SAMtools. Bioinformatics, 25, 2078–2079.
14. Hauenschild,R., Tserovski,L., Schmid,K., Thüring,K., Winz,M.-L., Sharma,S., Entian,K.-D., Wacheul,L., Lafontaine,D.L.J., Anderson,J., *et al.* (2015) The reverse transcription signature of N-1-methyladenosine in RNA-Seq is sequence dependent. Nucleic Acids Res., 43, 9950–9964.
15. Robinson,J.T., Thorvaldsdóttir,H., Winckler,W., Guttman,M., Lander,E.S., Getz,G. and Mesirov,J.P. (2011) Integrative genomics viewer. Nat. Biotechnol., 29, 24–26.
16. Brouillard,J.-N.P., Günther,S., Varma,A.K., Gryski,I., Herfst,C.A., Rahman,A.K.M.N., Leung,D.Y.M., Schlievert,P.M., Madrenas,J., Sundberg,E.J., *et al.* (2007) Crystal structure of the streptococcal superantigen SpeI and functional role of a novel loop domain in T cell activation by group V superantigens. J. Mol. Biol., 367, 925–934.
17. Broglia,L., Lécrivain,A.-L., Renault,T.T., Hahnke,K., Ahmed-Begrich,R., Le Rhun,A. and Charpentier,E. (2020) An RNA-seq based comparative approach reveals the transcriptome-wide interplay between 3′-to-5′ exoRNases and RNase Y. Nat. Commun., 11, 1587.
18. Walker,M.J., Hollands,A., Sanderson-Smith,M.L., Cole,J.N., Kirk,J.K., Henningham,A., McArthur,J.D., Dinkla,K., Aziz,R.K., Kansal,R.G., *et al.* (2007) DNase Sda1 provides selection pressure for a switch to invasive group A streptococcal infection. Nat. Med., 13, 981–985.
19. Broglia,L., Materne,S., Lécrivain,A.-L., Hahnke,K., Le Rhun,A. and Charpentier,E. (2018) RNase Y-mediated regulation of the streptococcal pyrogenic exotoxin B. RNA Biol., 15, 1336–1347.
20. Loh,J.M.S. and Proft,T. (2013) Toxin-antitoxin-stabilized reporter plasmids for biophotonic imaging of Group A *Streptococcus*. Appl. Microbiol. Biotechnol., 97, 9737–9745.
21. Silva-Rocha,R., Martínez-García,E., Calles,B., Chavarría,M., Arce-Rodríguez,A., de Las Heras,A., Páez-Espino,A.D., Durante-Rodríguez,G., Kim,J., Nikel,P.I., *et al.* (2013) The Standard European Vector Architecture (SEVA): a coherent platform for the analysis and deployment of complex prokaryotic phenotypes. Nucleic Acids Res., 41, D666-675.

22. Huang,Y., Lemieux,M.J., Song,J., Auer,M. and Wang,D.-N. (2003) Structure and Mechanism of the Glycerol-3-Phosphate Transporter from *Escherichia coli*. Science, 301, 616–620.

23. Wang,L. and Lutkenhaus,J. (1998) FtsK is an essential cell division protein that is localized to the septum and induced as part of the SOS response. Mol. Microbiol., 29, 731–740.

24. Dubarry,N. and Barre,F.-X. (2010) Fully efficient chromosome dimer resolution in *Escherichia coli* cells lacking the integral membrane domain of FtsK. EMBO J., 29, 597–605.

25. Yamaoka,J., Nakamura,E., Takeda,Y., Imamura,S. and Minato,N. (1998) Mutational Analysis of Superantigen Activity Responsible for the Induction of Skin Erythema by Streptococcal Pyrogenic Exotoxin C. Infect. Immun., 66, 5020–5026.

26. Roussel,A., Anderson,B.F., Baker,H.M., Fraser,J.D. and Baker,E.N. (1997) Crystal structure of the streptococcal superantigen SPE-C: dimerization and zinc binding suggest a novel mode of interaction with MHC class II molecules. Nat. Struct. Biol., 4, 635.

27. Rush,J.S., Parajuli,P., Ruda,A., Li,J., Pohane,A.A., Zamakhaeva,S., Rahman,M.M., Chang,J.C., Gogos,A., Kenner,C.W., *et al.* (2022) PplD is a de-N-acetylase of the cell wall linkage unit of streptococcal rhamnopolysaccharides. Nat. Commun., 13, 590.

28. Chu,B.C.H., DeWolf,T. and Vogel,H.J. (2013) Role of the Two Structural Domains from the Periplasmic *Escherichia coli* Histidine-binding Protein HisJ. J. Biol. Chem., 288, 31409–31422.

29. Zhang,Y., Gonzalez-Gutierrez,G., Legg,K.A., Walsh,B.J.C., Pis Diez,C.M., Edmonds,K.A. and Giedroc,D.P. (2022) Discovery and structure of a widespread bacterial ABC transporter specific for ergothioneine. Nat. Commun., 13, 7586.

30. Christiansen,C., Abou Hachem,M., Janeček,Š., Viksø-Nielsen,A., Blennow,A. and Svensson,B. (2009) The carbohydrate-binding module family 20 - diversity, structure, and function: Carbohydrate-binding module family 20. FEBS J., 276, 5006–5029.

31. Janeček,Š., Svensson,B. and MacGregor,E.A. (2011) Structural and evolutionary aspects of two families of non-catalytic domains present in starch and glycogen binding proteins from microbes, plants and animals. Enzyme Microb. Technol., 49, 429–440.

32. Hartmann,M.D., Bourenkov,G.P., Oberschall,A., Strizhov,N. and Bartunik,H.D. (2006) Mechanism of Phosphoryl Transfer Catalyzed by Shikimate Kinase from *Mycobacterium tuberculosis*. J. Mol. Biol., 364, 411–423.

33. Gullett,J.M., Cuypers,M.G., Frank,M.W., White,S.W. and Rock,C.O. (2019) A fatty acid-binding protein of *Streptococcus pneumoniae* facilitates the acquisition of host polyunsaturated fatty acids. J. Biol. Chem., 294, 16416–16428.

34. Shi,Y., Zang,N., Lou,N., Xu,Y., Sun,J., Huang,M., Zhang,H., Lu,H., Zhou,C. and Feng,Y. (2022) Structure and mechanism for streptococcal fatty acid kinase (Fak) system dedicated to host fatty acid scavenging. Sci. Adv., 8, eabq3944.
35. Jung,W.-S., Singh,R.K., Lee,J.-K. and Pan,C.-H. (2013) Crystal Structure and Substrate Specificity of D-Galactose-6-Phosphate Isomerase Complexed with Substrates. PLoS ONE, 8, e72902.
36. Loughman,J.A. and Caparon,M.G. (2007) Comparative functional analysis of the *lac* operons in *Streptococcus pyogenes*. Mol. Microbiol., 64, 269–280.
37. Sharma,A., Kottur,J., Narayanan,N. and Nair,D.T. (2013) A strategically located serine residue is critical for the mutator activity of DNA polymerase IV from *Escherichia coli*. Nucleic Acids Res., 41, 5104–5114.
38. Cheng,G., Bennett,E.M., Begley,T.P. and Ealick,S.E. (2002) Crystal Structure of 4-Amino-5-Hydroxymethyl-2-Methylpyrimidine Phosphate Kinase from *Salmonella typhimurium* at 2.3 Å Resolution. Structure, 10, 225–235.
39. Cea,P.A., Araya,G., Vallejos,G., Recabarren,R., Alzate-Morales,J., Babul,J., Guixé,V. and Castro-Fernandez,V. (2020) Characterization of hydroxymethylpyrimidine phosphate kinase from mesophilic and thermophilic bacteria and structural insights into their differential thermal stability. Arch. Biochem. Biophys., 688, 108389.
40. Newberry,K.J. (2002) Structural origins of amino acid selection without editing by cysteinyl-tRNA synthetase. EMBO J., 21, 2778–2787.
41. Hauenstein,S., Zhang,C.-M., Hou,Y.-M. and Perona,J.J. (2004) Shape-selective RNA recognition by cysteinyl-tRNA synthetase. Nat. Struct. Mol. Biol., 11, 1134–1141.
42. Crepin,T., Yaremchuk,A., Tukalo,M. and Cusack,S. (2006) Structures of Two Bacterial Prolyl-tRNA Synthetases with and without a cis-Editing Domain. Structure, 14, 1511–1525.
43. Vey,J.L., Yang,J., Li,M., Broderick,W.E., Broderick,J.B. and Drennan,C.L. (2008) Structural basis for glycyl radical formation by pyruvate formate-lyase activating enzyme. Proc. Natl. Acad. Sci., 105, 16137–16141.
44. Metz,A.H., Hollis,T. and Eichman,B.F. (2007) DNA damage recognition and repair by 3-methyladenine DNA glycosylase I (TAG). EMBO J., 26, 2411–2420.
45. Zhu,X., Yan,X., Carter,L.G., Liu,H., Graham,S., Coote,P.J. and Naismith,J. (2012) A model for 3-methyladenine recognition by 3-methyladenine DNA glycosylase I (TAG) from *Staphylococcus aureus*. Acta Crystallogr. Sect. F Struct. Biol. Cryst., 68, 610–615.

46. Choi,Y., Sims,G.E., Murphy,S., Miller,J.R. and Chan,A.P. (2012) Predicting the Functional Effect of Amino Acid Substitutions and Indels. PLoS ONE, 7, e46688.
